# Kings and peasants from El-Zuma/El-Detti microregion in the Early Makurian period. Economic aspects of animal bones from funerary contexts

**DOI:** 10.1371/journal.pone.0212423

**Published:** 2019-02-15

**Authors:** Urszula Iwaszczuk, Justyna Niderla-Bielińska, Aneta Ścieżyńska

**Affiliations:** 1 Polish Centre of Mediterranean Archaeology, University of Warsaw, Warsaw, Poland; 2 Histology and Embryology Department, Medical University of Warsaw, Warsaw, Poland; Seoul National University College of Medicine, REPUBLIC OF KOREA

## Abstract

Tumuli fields at El-Zuma and El-Detti were dated to the 2nd phase of the Early Makurian period, *c*. AD 450–550. They represented three types of tombs of different sizes and structures. The animal remains from these graves were analyzed in the context of animal economy practiced by the people who lived in the vicinity of the burial sites. aDNA analysis was conducted for cattle remains to explain its origin and significance for the inhabitants of the region. The research showed agricultural nature of the settlement located to the north of the Nile Valley with a great importance of cattle and sheep breeding. It also indicated the northern direction of trade and cultural contacts of the society based in the El-Zuma/El-Detti microregion and the deep social stratification within this group.

## Introduction

Tumuli excavated at archaeological sites in El-Zuma and El-Detti, Sudan, are dated to the Early Makurian period. The only investigated archaeological sites dated to that period are cemeteries. The absence of known settlements is a particular problem for researchers, which makes the examination of everyday life of the people who lived there virtually impossible. There is but a minor chance for archaeologists to discover and examine settlements in the area adjacent to the Nile because of the scarcity of suitable territories for habitation. Such conditions force occupation of the same places for generations and, in consequence, accumulation of settlement activity in the area which persists today. That is why it is important to look for other sources of information concerning economy, trade contacts and other kinds of activities in the Early Makurian period.

Tumuli at El-Zuma have been excavated for twelve years (seasons 2005, 2007, 2009, 2011, 2012, 2013, 2014, 2015 and 2017). Some of the results were published by M. El-Tayeb [1,2], M.El-Tayeb & E. Czyżewska [3] and M. El-Tayeb et al. [4], yet it seems that there is still very little known about the people who buried their deceased at the cemetery. The bone material from two seasons of excavations was analyzed by M. Osypińska [5, 6]. It is interesting that M. Osypińska, who saw the animal bone remains in 2007 and 2009, stated in her articles that the bones were in good condition and most of them complete. In 2013 the majority of the bones were in a poor state of preservation, very fragmented or crumbled into shards before they could be identified and measured [7]. A similar situation concerns the animal remains from the El-Detti tumuli field excavated in 2015 [8]. Because of a such state of preservation and due to the presence of a high number of bones that came from young animals there is only a very small assemblage of bones that can be measured. That also requires other techniques than archaeozoological ones to try to establish the morphotypes of the animals discovered in the tumuli. One such technique is genetic analysis. There have been some successes in this field over the last twenty years in the case of African animals. Although most authors analyzing aDNA of cattle concentrated on the domestication process [9, 10, 11], other researchers became interested in analyzing aDNA from Sudanese excavations [12]. Genetic research in Africa is still performed very seldom, especially with regards to Sudan—studies on animal aDNA have not yet been provided. The project by the University in Khartoum concerned only human remains, but it shows that general approach has changed (http://www.worldfamilies.net/forum/index.php?topic=11097.0;wap2). African continent has much to offer to archaeologists, archaeozoologists and genetics researchers, yet such interdisciplinary projects are carried out very seldom. Such an approach is unique as researchers in general decide to use animal remains from funerary contexts only to reconstruct funerary rituals.

The main goal of this paper is an attempt to discover facts concerning economy of the El-Zuma/El-Detti microregion on the basis of animal bone remains from tumuli dated to the Early Makurian period by means of two-stage research: conventional archaeozoological examination and aDNA analysis of cattle remains from El-Zuma and El-Detti tumuli fields.

## Material and methods

### Archaeozoological analysis

Bone material analyzed in this paper came from two tumuli fields at El-Zuma (18°22′09,69″N 31°44′19.31″E) and El-Detti (18°25′27.41″N 31°46′33.67″E), situated on the right bank of the Nile between the 3^rd^ and 4^th^ Cataracts, both excavated by Mahmoud El-Tayeb within the framework of the Early Makuria Research Project of the Polish Centre of Mediterranean Archaeology of the University of Warsaw. Both archaeological sites were dated to the 2nd phase of the Early Makurian period, c. AD 450–550 [13].

The research data was collected in the course of excavations carried out in seasons 2009–2017. Archaeological analysis of the animal remains from tumuli 15 and 21 at El-Zuma and seven tumuli at El-Detti and the ritual aspect of these remains has already been partially discussed [7, 8]. Additionally, bone fragments from tumuli 2, 5, 10 and 25 from El-Zuma had been analyzed by M. Osypińska [6, 14] and therefore they were not included in the present research. The bone material from all analyzed tumuli from both archaeological sites was identified to a zoological taxon and anatomical element. The age of the individuals was estimated on the basis of the fusion of long bone epiphyses with shafts, the fusion of pelvis bones and the degree of development of the glenoid articulation of scapula as well as on the basis of tooth development [15, 16]. When it was possible, ovicaprine remains were identified according to standard methods [17, 18, 19]. The bones were measured according to the Driesch’s method [20]. All the measurements were listed in Table 1. Height at the withers was estimated for some cattle and sheep individuals, on the basis of Teichert’s coefficients for sheep [21], Matolcsi’s coefficients for cattle radius and tibia [21] and Fock’s coefficient for cattle metatarsal [22]. The number of individuals (MNI) was calculated, taking into consideration the size of the bones and the age and sex of the individuals if they were possible to establish.

**Table 1:** Measurements of animal bones from El-Zuma.

Tumulus 3			Tumulus 4					Tumulus 14		
CATTLE			CATTLE					CATTLE		
Scapula			Scapula	Humerus	Radius	Metacarpal	Pelvis	Scapula		
GLP	BG	SLC	SLC	Bd	Bd	Bd	LA	GLP	BG	SLC
				82	65.7	48.3	56.3	76.2	51.7	61
58.4	51	57	56.8	80.2		59.4		**Femur**		
			47.2	63		47		Bd		
**Humerus**			**Femur**		**Tibia**			100.4		
Bd			Bp	Bd	GL	Bd		**Talus**		
			120.3			55.9		GLl	GLm	Bd
70.5				/85/	349	59.3		73.8	67.2	46.5
						57.3		**SHEEP**		
**Radius**			**Talus**					**Humerus**	**Radius**	
Bp	Bd	SD	GLl	GLm		Bd		Bd	Bp	Bd
			64.9	61.9		41.4		29	29.9	28.5
84.5	77.7	44.8	68.7	60		43		**Femur**		
			64	59.5		41.5		GL	Bp	Bd
**Tibia**			64.5	59.6		40.8		174	39.4	35
Bp	Bd	SD	67.4	62.9		44.8		**Tibia**		
			**Metatarsal**					Bp	Bd	
	54.6		GL	Bp	Bd	SD	DD	38.8	26.4	
				46.3	54	25.4	27.8	38.9	26.7	
95	58.2	41.3	233	40.4	45.3	23	23.8	**Talus**		
			**Ph I**					GLl	GLm	Bd
**SHEEP**			GL	Bp	Bd	SD		29.6	28.2	18.4
**Humerus**	**Pelvis**		72	30.8	28	27		29.8	28.3	19
Bd	LA		69.7	28	27.6	25.5		**Tumulus 15**		
			**DONKEY**					**CATTLE**		
32.5	22.8		**Pelvis**	**Ph I**				**Scapula**		**Tibia**
			LA	GL	Bp	Bd	SD	GLP	SLC	Bd
**Tumulus 7**			46.8	67	33.2	31.2	23	71.3	64.8	64.2
**CATTLE**			**Radius**				**Femur**	**Talus**		
**Talus**			GL	Bp	Bd	SD	Bp	GLl	GLm	Bd
GLl	GLm	Bd	267	59	50.5	27	83.2	70.2		46
69.8	63.1	45.2	**CAMEL**					69.8		47
57.2	54.5		**Scapula**					69.2	59.6	46.2
**Metacarpal**			GLP	BG		SLC		69.1	60	46.2
Bp			105	57		72		**SHEEP**		
			**Humerus**		**Metacarpal**			**Humerus**		
47.2			Bd		Bp			Bd		
			87.8		59			32.2		

**Fig 1:**
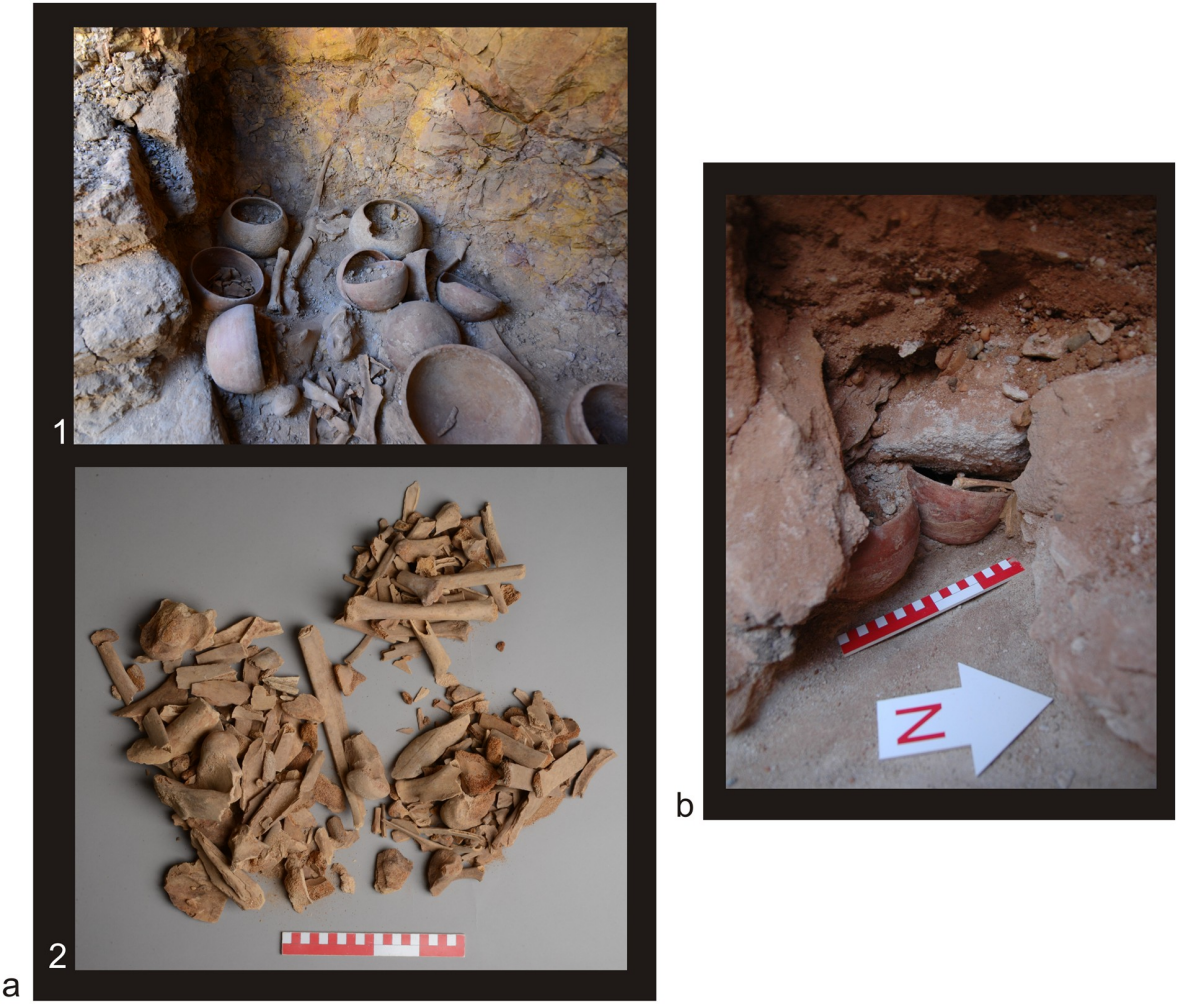
State of preservation of the animal remains from the El-Zuma tumuli field (Phot. Adam Kamrowski). Tumulus 26, chamber 2, southern part: **a1**. after removing the blockage, **a2**. animal bones delivered to the analysis; **b**. Tumulus 21, the chamber with a collapsed ceiling.

**Fig 2:**
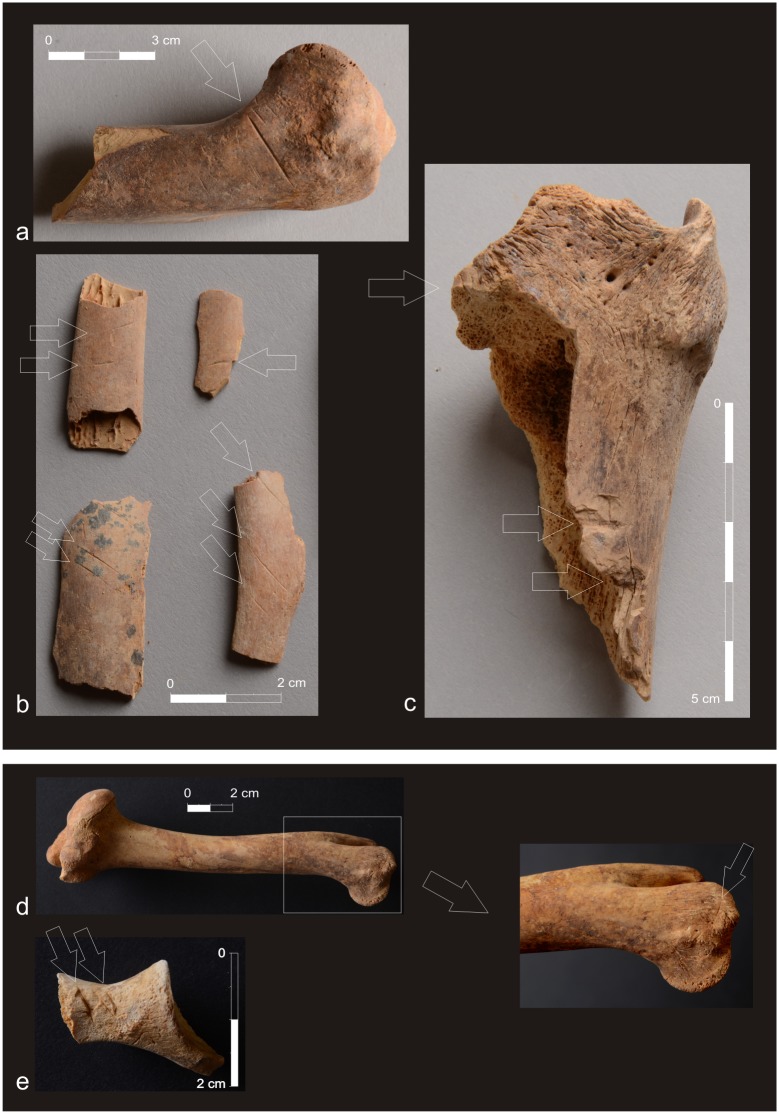
El-Zuma tumuli field, tumulus 26, chamber 2: **a1**. marks of skinning visible on sheep humerus, **a2**. marks of filleting visible on the sheep/goat ribs, **a3**. marks of chopping visible on cattle tibia; El-Detti tumuli field: **b1**. marks of skinning visible on sheep humerus (tumulus 4, chamber), **b2**. marks of cutting visible on sheep/goat pelvis (tumulus 2, chamber) (Phot. Adam Kamrowski).

The state of preservation of analyzed bones differed. Generally, assemblages from El-Zuma tumuli field were in a much poorer state of preservation than the remains from El-Detti, which, among others, was probably the result of the flood that took place in 2009. The graves at El-Zuma are much deeper than those at El-Detti, so the water must have been accumulated there much longer than in the shallow graves at El-Detti. The climate conditions in this part of Sudan are rather harsh and the high temperatures must have also had their share in the deterioration of the animal remains. Some of the bones were weathered, very dry and fragile and in consequence they were broken into many pieces as a result of the exploration of the graves (Fig 1). Hot climate also caused further destruction of the remains after the exploration. Bones, that had been waiting for the analysis in the storeroom for some years, were in much worse state of preservation than freshly excavated ones. The destruction of the surface of the bones was also deeper, the degree of the fragmentation was higher as well. Some of the bones bore post-consumption marks: marks of skinning, filleting, chopping (Fig 2).

### aDNA analysis

#### Sample collection and DNA isolation

Samples were collected on site and immediately packed into sterile falcon tubes to minimize the risk of cross contamination. 55 samples from the collected material were further processed for DNA isolation (S1 Table), including 14 fragments of humeri, 13 of tibiae, 3 of pelvises, 9 of femurs, 1 of radius, 4 of scapulae, 2 of skulls, 6 of teeth, 1 of metatarsus, 1 of ulna and 1 of metacarpus (compact bone or dentin was taken for isolation). All samples were processed under strict rules for contamination prevention, including separation of pre-PCR and post-PCR labs, cleaning of surfaces and equipment with 8% bleach, and UV-irradiation of rooms, laboratory hoods and equipment. DNA was isolated according to the protocol described by Rohland and Hofreiter [23] with some modifications. Samples were first cleaned and exposed to UV light for 60’ to reduce the cross contamination risk and pulverized with a hand mortar and pestle. Then samples of 500 mg of powder each were suspended in 10 ml of Extraction Buffer (0.2 mg/ml Proteinase K in 0.45 M EDTA, pH 8.0) and incubated overnight in 37 °C with gentle agitation. Blank extraction, without bone powder, was also included. Next day, after centrifugation the supernatant was transferred into a 50 ml conical tube and mixed with 40 ml of Binding Buffer (5 M GuSCN, 25 mM NaCl, 50 mM TrisBase). Then 100 μl of silica beads suspension was added to the mixture and pH was adjusted to 4.0 with 30% HCl. Suspension was incubated in room temperature, in the dark for 3 hours with gentle agitation. After incubation the suspension was centrifuged, the supernatant discarded and the pellet resuspended in 1 ml Binding Buffer. Next, the suspension was transferred into 1.5 ml tube, centrifuged and pellet was washed twice with Washing Buffer (50% ethanol, 125 mM NaCl, 10mM TrisBase, 1mM EDTA, pH 8.0). After last centrifugation the remaining Wash Buffer was removed and the pellet was dried at room temperature. Then pellet was resuspended in 50 μl of prewarmed TE buffer (10mM TrisHCl, 1 mM EDTA, pH 8.0) and incubated for 10’ on the thermoshaker in 55 °C with gentle agitation. Finally, the suspension was centrifuged and the supernatant, containing DNA, carefully transferred to a clean 1.5 ml tube. DNA concentration was measured with NanoDrop spectrophotometer. The samples were stored at -20 °C.

#### Amplification of mitochondrial d-loop sequence

DNA was amplified with four primer sets, mapping d-loop of mitochondrial DNA from 16651 to 433 position. Primers were designed according to the paper published by Kim et. al. [24] and reference sequence GenBank: V00654.1 to cover the targeted region in four overlapping fragments: (1) forward primer–GACAGGTCTTTGTAGTACAT, reverse primer GTAATTCATTCTGTGGTCTGTG, product length 298 bp; (2) forward primer–CACAGACCACAGAATGAATTAC, reverse primer–GCCCGGAGCGAGAAGAGGGA, product length 296 bp; (3) forward primer–TCCCTCTTCTCGCTCCGGGC, reverse primer–CATTATGCTGGTGCTCAAGATGC, product length 297 bp; (4) forward primer–GCATCTTGAGCACCAGCATAATG, reverse primer–ATGTGTTTATGGAGTTGGGA, product length 294 bp. Blank controls were included during each PCR. PCR was performed with KAPA2G Robust PCR Kit according to the producer’s protocol with 0.5 U KAPA2G Robust DNA polymerase (KAPA Biosystems), 1x KAPA2G buffer (KAPA Biosystems), 3 mM MgCl2 (KAPA Biosystems), 0.2 mM dNTP’s (TaKaRa Bio.), 1 X KAPPA Enhancer (KAPA Biosystems), 0.2 μM primer (DNA, IBB, PAN, Poland) with addition of PCR Anti-inhibitor (RP50, DNA Gdansk, Poland) and 25 ng of DNA template. Amplification was carried out in a Mastercycler gradient (Eppendorf); cycling parameters were: denaturation—95°C/30 sec, annealing—50°C/30 sec and elongation—72°C/30 sec for 50 cycles. Elongation time was extended for 5 sec every 5 cycles. PCR products were visualized on an 1.5% agarose gel by addition of Midori Green Direct DNA Stain, appropriate bands were excised and purified from the gel with EXTRACTME DNA GEL-OUT KIT (EM08, DNA Gdansk, Poland). Sequencing products were obtained using the BigDye Terminator v.3.1 chemistry (Applied Biosystems). Products were sequenced by capillary electrophoresis on an ABI PRISM 3500 Genetic Analyzer (Applied Biosystems) using POP-7 polymer (Applied Biosystems).

### Phylogenetic analysis of sequences

Obtained sequences were analyzed with Phylogeny.fr platform with the advanced mode and with MEGA7 software. Programs used for analysis were: alignments—MUSCLE 3.7, ClustalW, phylogeny—PhyML 3.0 and for tree rendering—TreeDyn 198.3 [25, 26, 27, 28, 29]. Obtained sequences were aligned with published sequences of various *Bos* breeds from Africa; *Bos indicus* (Hariana and Zebu) sequences were used as an outgroup. For details see Table 2.

**Table 2:** Origin of mtDNA fragments from various cattle breeds compared in this study.

GenBank accesion no.	Breed/sample no.	Country	Reference
EF524185	ZEBU	India	unpublished
AB085923	HARIANA	India	[30]
FJ440734	RAYA-AZEBO	Ethiopia	[31]
FJ440833	DANAKIL	Ethiopia	[31]
FJ440830	DANAKIL	Ethiopia	[31]
FJ440808	ADWA	Ethiopia	[31]
FJ440788	HORRO	Ethiopia	[31]
FJ440747	BORAN_1	Ethiopia	[31]
FJ440754	BORAN_2	Ethiopia	[31]
FJ440802	OGADEN	Ethiopia	[31]
FJ440772	AMBO	Ethiopia	[31]
FJ440762	ARSI	Ethiopia	[31]
FJ440825	FOGERA_1	Ethiopia	[31]
FJ440821	FOGERA_2	Ethiopia	[31]
L27720	WHITE-FULANI_1	Africa, Sahel	[10]
L27721	WHITE-FULANI_2	Africa, Sahel	[10]
JN817298	ABIGAR	Ethiopia	[32]
EU747737	WATUSI	East Africa	unpublished
FJ440732	SHEKO_1	Ethiopia	[31]
FJ440726	SHEKO_2	Ethiopia	[31]
KT184455	MENOFI_1	Egypt	[33]
KT184453	MENOFI_2	Egypt	[33]
KT184451	MENOFI_3	Egypt	[33]
KT184461	MENOFI_4	Egypt	[33]
KT184459	MENOFI_5	Egypt	[33]
KT184456	MENOFI_6	Egypt	[33]
KT184452	DOMIATY_1	Egypt	[33]
KT184458	DOMIATY_2	Egypt	[33]
KT184460	DOMIATY_3	Egypt	[33]
KT184457	DOMIATY_4	Egypt	[33]
L27728	KENANA	Kenya	[10]
L27714	BUTANA	Kenya	[10]
MG892465	SUDAN_EL_ZUMA_ZS39	Sudan	This paper
MG892466	SUDAN_EL_DETTI_DS3	Sudan	This paper

## Results

Full analysis of animal offerings from El-Zuma and El-Detti tumuli fields as a part of funerary rituals is currently in preparation as a part of a monograph concerning El-Zuma site. All the animal bones excavated in 2009–2017 were identified by the first author in the course of field works in seasons 2013–2017, but for the clarity of the reasoning only the examples of typical and untypical assemblages will be discussed below.

### EL-ZUMA tumuli field

**Fig 3:**
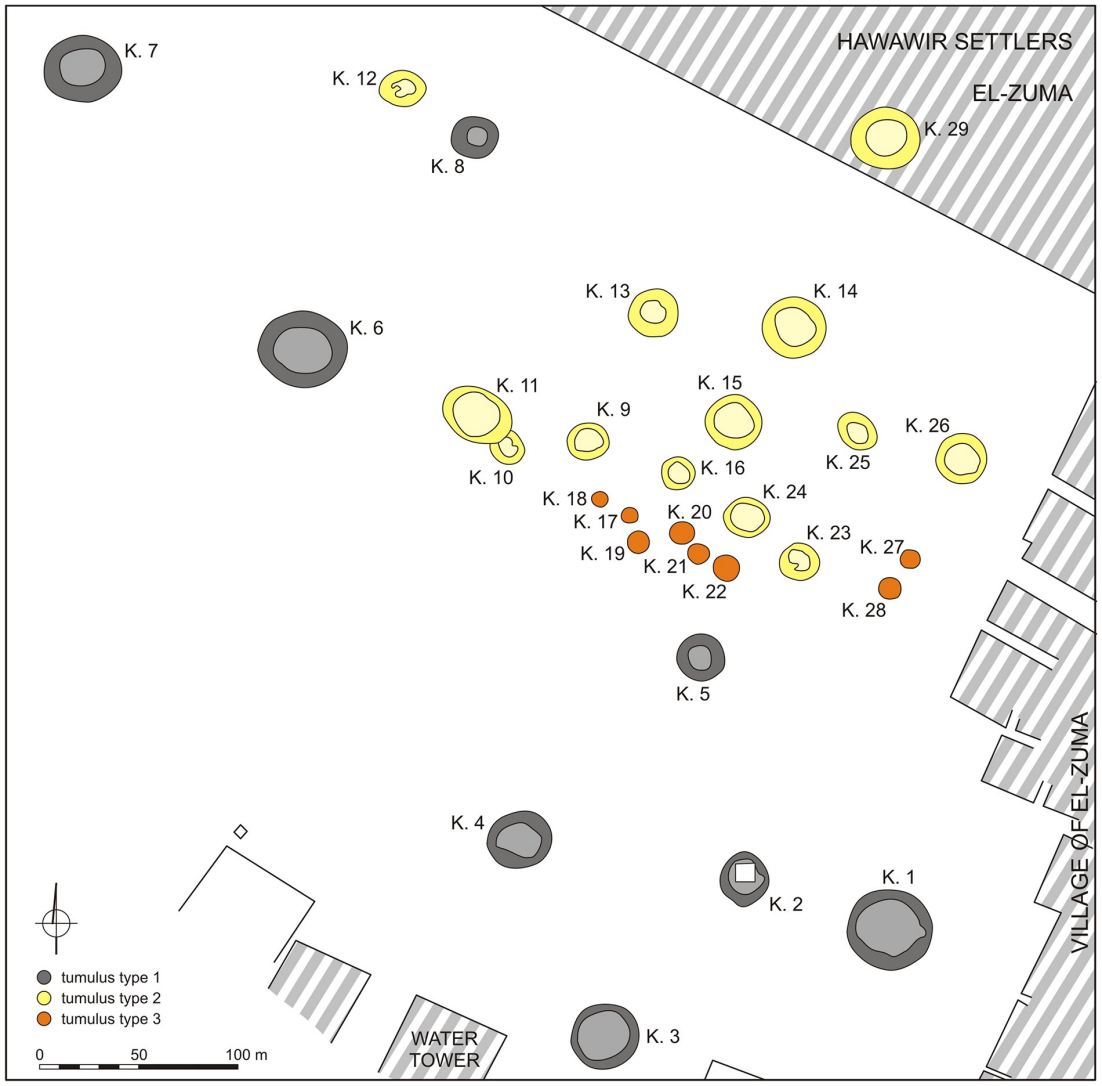
Plan of the El-Zuma tumuli field (Copyright Makuria Research Project, Polish Centre of Mediterranean Archaeology of the University of Warsaw).

The tumuli field at El-Zuma (Fig 3) is located on the edge of the village, about 2 km from the present day Nile valley and about 20 km downstream from the town of Karima, Sudan. It is one of the biggest sites of this type and the only one in the region of Dongola Reach that has been excavated in such a great degree. The regular excavations in El-Zuma have been carried on since 2005 [1, 2, 3, 4, 34]. The tumuli were classified by Artur Obłuski [35] into three categories on the basis of the differences in superstructures and later on this classification was confirmed by Mahmoud El-Tayeb [2, 4], who also analyzed their substructures. Tumuli of type 1 were the biggest structures of the site. Their mounds of conical shape were built of a mixture of earth and gravel, fully coated with rough black stones. They had the most complex substructures, which consisted of a U-shaped shaft in the central part of the tomb, the main burial chamber cut into the southern wall of the shaft and side chambers of different numbers and shapes hewn into the western and northern sides of the grave. The most characteristic feature of this type of tombs was an underground tunnel of unknown function leading in the direction of the main burial chamber. Tumuli of type 2 were usually smaller than tumuli of type 1 and they differed from them in one vital point: they did not contain tunnels. They also had different shape of the superstructures in the form of flat-top mounds. The smallest tumuli, of type 3, also had flat mounds built of a mixture of earth, sand and gravel that were surrounded by stone rings on the ground surface. They contained only a rectangular shaft and one burial chamber cut usually in the western wall of the shaft. Unfortunately all the tombs had been looted in the past. The traces of robbers’ activity were usually noted on the surface of the mounds and in the main chambers, especially in the vicinity of the deceased, while the side chambers were left untouched with all their contents *in situ*. Animal remains, together with other artifacts, were discovered directly on the chambers’ floors and in the fill of the shafts and tunnels. The bones were also found in the robber’s holes but these were probably not connected with the funeral rituals and got there accidentally during natural processes.

The animal bones discussed below were present in different numbers in all the excavated tombs but two: tumulus 18 and tumulus 27 lacked the animal material entirely. The remains were found in all the structures of the tombs, that is in main chambers, side chambers, shafts and tunnels with shafts leading to them.

#### Tumuli of type 1

There are 8 tumuli of type 1 at El-Zuma still visible on the surface, numbered from 1 to 8 (Fig 3). They are located in the southernmost and north-western parts of the site. Bone assemblages from tumuli 2 and 5 of type 1 excavated in seasons 2005 and 2007 were analyzed by Marta Osypińska [14, 6]. Only one of the remaining tumuli was excavated in a greater degree (tumulus 3), in the case of others only tunnels were explored.

The offerings situated in the chambers of tumuli 2, 3, 4, 5 and 7 were covered by the fragments of the stone ceiling. Because of the collapsing of the ceiling a part of the animal remains was fragmented. The fill of the tunnels varied. They usually contained layers of sand and gravels of various thickness, rock fragments from the walls and ceilings of the tunnels. Initial analysis of the pottery shards from the tunnels of tumuli of type 1 showed that the pieces of the same vessels were found in different layers (E. Czyżewska-Zalewska, personal communication). It seems that natural processes (flood, deposition of the fill of the shaft in the tunnels) and robber’ activity were the cause of the accumulation of different layers in the tunnels of tumuli of type 1.

Bone assemblages from three excavated tumuli of type 1 will be presented below: the best examined tumulus 3 and partially excavated tumulus 7 as the representatives of the typical grave offerings and tumulus 4, exceptional in many ways, which will be discussed further.

For a DNA research a set of samples was collected from cattle bones from the following tumuli: tumuls 1 (maxillary tooth M3, mandibular tooth M2, ulna, femur, 3 tibias, metacarpal), tumulus 3 (humerus), tumulus 4 (2 fragments of skull, maxillary tooth M1, mandibular tooth P4, 2 mandibular teeth M3, 2 femurs, tibia, metatarsal), tumulus 8 (femur, tibia).

**Fig 4:**
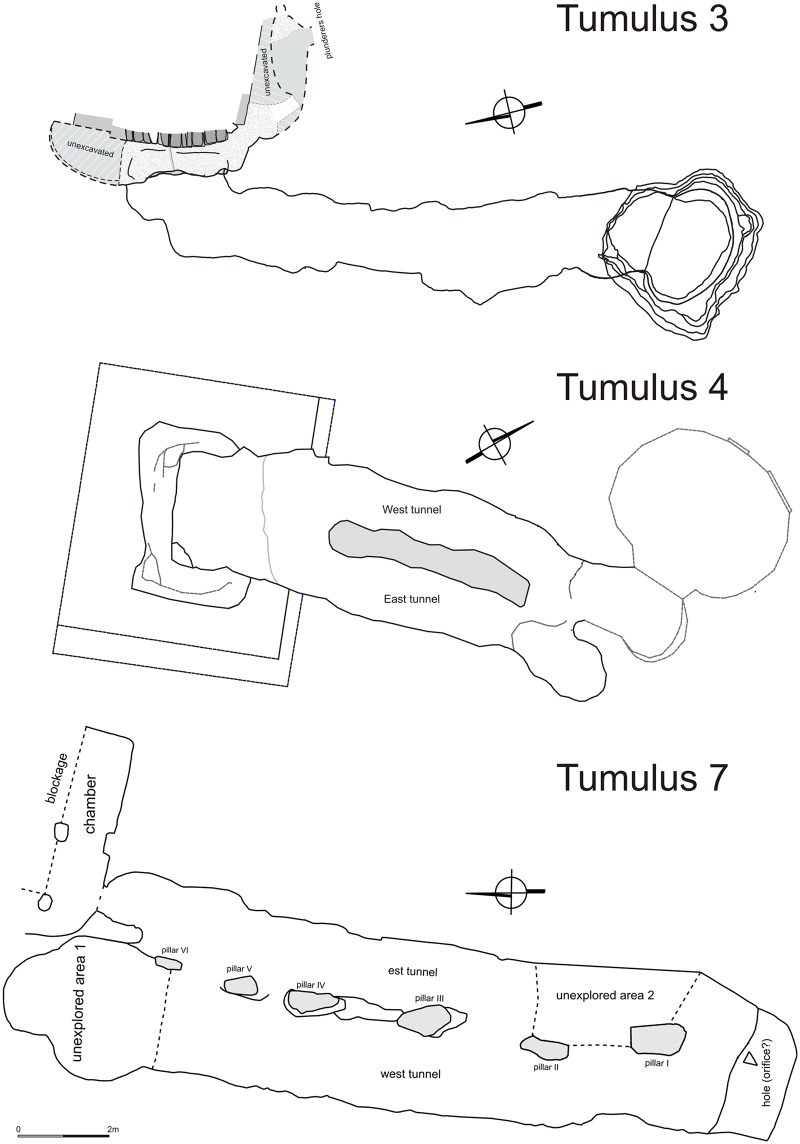
El-Zuma. Plans of tumuli of type 1: tumulus 3 (draw. and digit. J. Juchniewicz), tumulus 4 (draw. M. Antos), tumulus 7 (draw. K. Juchniewicz, digit. E. Czyżewska).

**Tumulus 3** was excavated in 2014. Its superstructure was about 4 m high with a diameter of about 30 m. The archeological team entered through the tunnel which turned out to be almost 12 m long. It was possible to examine partially the main burial chamber (chamber 1) and one of the side chambers (chamber 2) (Fig 4). Unfortunately all the chambers had been looted, the archaeological material, i.e. pottery and beads as well as human and animal remains were mixed together. It seems that some offerings located originally in chamber 1 and probably also in chamber 2 were thrown to the tunnel during the grave robbery.

**Fig 5:**
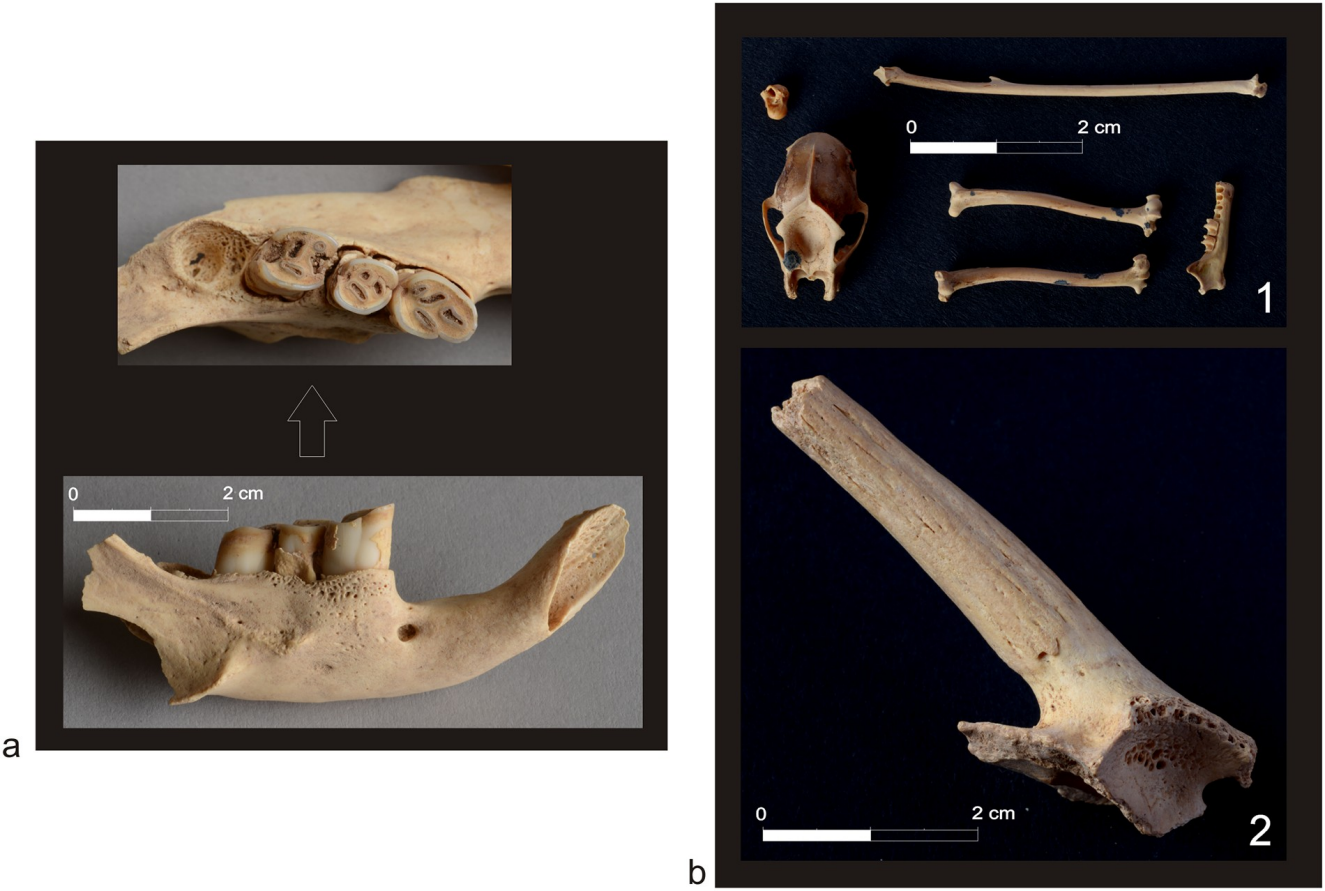
Bones of wild mammals found in the tunnels of tumuli of type 1 (Phot. Adam Kamrowski). **a**. mandible of porcupine (tumulus 3); **b1**. some remains of bat (tumulus 4), **b2**. horn core of female *Gazella Dorcas*.

The animal remains from that tomb were found mostly in one layer and were relatively numerous. It was possible to identify 336 bone fragments, 45 fragments remained unidentified. The majority of the bones came from cattle and sheep. Some of these bones bore post-consumption marks. Except for these two species there were also remains of porcupine (Fig 5), dog, micro-mammals (in size of mouse) and a bird in a pigeon size—almost all of them in the tunnel and the fill of the robbery trench, only 2 micro-mammal bone fragments were located in chamber 1 (Table 3).

**Table 3:** Animal bones from tumulus 3.

Species	Localization	Bone	Number of fragments (NISP)	Number of bones (MNE)			Description (MNI, age, additional information)
				Right	Left	Right/Left	
Cattle	Chamber 1	Rib	16			1	1 individual: under 3.5 years old
	Chamber 2	Tooth	18			1	
		Rib	4			3	
		Humerus	6		1		
		Femur	2			1	
		Patella	1			1	
	Niche	Rib	2			1	2 individuals: 1 under 3.5–4 years old; 1 adult individual
		Humerus	1			1	
	Tunnel	Mandible	16	1			
		Sternum	7			3	
		Rib	31			3	
		Scapula	6			1	
		Humerus	3		1		
		Radius	1		1		
		Ulna	1		1		
		Ossa carpi	1			1	
		Metacarpal	21		1		
		Pelvis	19	1	1		
		Femur	12	1	1		
		Patella	1			1	
		Tibia	1		1		
		Centroquartale	2			1	
		Metatarsal	2	1			
	Robbery trench	Rib	2			1	adult individual or individuals
		Scapula	22		1		
		Tibia	2		1		
		Ph III	1		1		
Sheep	Chamber 1	Sternum	29			7	1 individual: about 3.5 years old
		Rib	43			4	
		Scapula	9	1			
		Humerus	11	1			
	Tunnel	Pelvis	2		1		adult individual or individuals
		Femur	5		1		
Ovicaprine	Tunnel	Scapula	2			1	adult individual or individuals
		Humerus	3			1	
	Robbery trench	Rib	3			1	
Dog	Robbery trench	Humerus	9		1		adult individual or individuals
		Ulna	1		1		
		Femur	1		1		
		Calcaneus	1		1		
		Metatarsal	1		1		
Porcupine	Tunnel	Mandible	7	1			old individual
Micro-mammal	Chamber 1	Femur	1	1			adult individuals
		Tibia	1	1			
	Tunnel	Tibia	2	2			
Bird	Tunnel	Vertebra	2			2	adult individual: pigeon size
		Coracoid	2			1	

Cattle bones found in all the explored structures had fallen into pieces (201 fragments came from only 35 bones). The remains from proximal parts of the forelimb (humerus) and hind limb (femur, patella) with an addition of four ribs and a tooth deposited in chambers 1 and 2 came from a single individual under 3.5 years old. There were also much more numerous cattle bone fragments in the tunnel. A lot of them came from the proximal parts of the forelimb (scapula, humerus, radius, ulna) and hind limb (pelvis, femur, patella, tibia), mostly from the left side of the carcass, but some bone elements belonged to the trunk (ribs and sternum), head (mandible) and distal parts of the forelimb (ossa carpi, metacarpals) and hind limb (centroquartale, metatarsal) from both sides of the carcass. Additionally, three bone fragments from a rib and humerus were found in the niche that was cut in the tunnel. The remains from the tunnel belonged to two individuals: one was under 3.5–4 years old, the other was adult. Another four cattle bones (rib, scapula, tibia, phalanx III) that came from an adult animal or animals and were found in the fill of the robbery trench were probably accidental depositions and unconnected with the burial ritual.

Sheep bones (99 fragments from 18 bones) were located in two contexts: chamber 1 and the tunnel. All of them came from the parts of the carcass with good quality meat, such as trunk (sternum, ribs) and proximal parts of the right forelimb (scapula, humerus) and left hind limb (pelvis, femur). They belonged to two individuals—one about 3.5 years old and probably one adult animal. Further three ovicaprine bones were found in the tunnel (scapula, humerus) and in the fill of the robbery trench (rib).

Adult dog remains were few (13 fragments from 5 bones) and all of them were found in the fill of the robbery trench, which means they might have been unconnected with the burial ritual. 7 fragments of one mandible of an old porcupine are much more puzzling. They were found in the tunnel which might suggest that they could have been left there (or in one of the chambers) during the funerary ritual but not necessarily—the porcupine bone might have got there when the shaft leading to the tunnel was buried. The bones of micro-mammals (probably a kind of mouse) and a pigeon-size bird were much less surprising if one takes into consideration that the tunnel was left open for some time either after the funeral or after the robbery.

**Fig 6:**
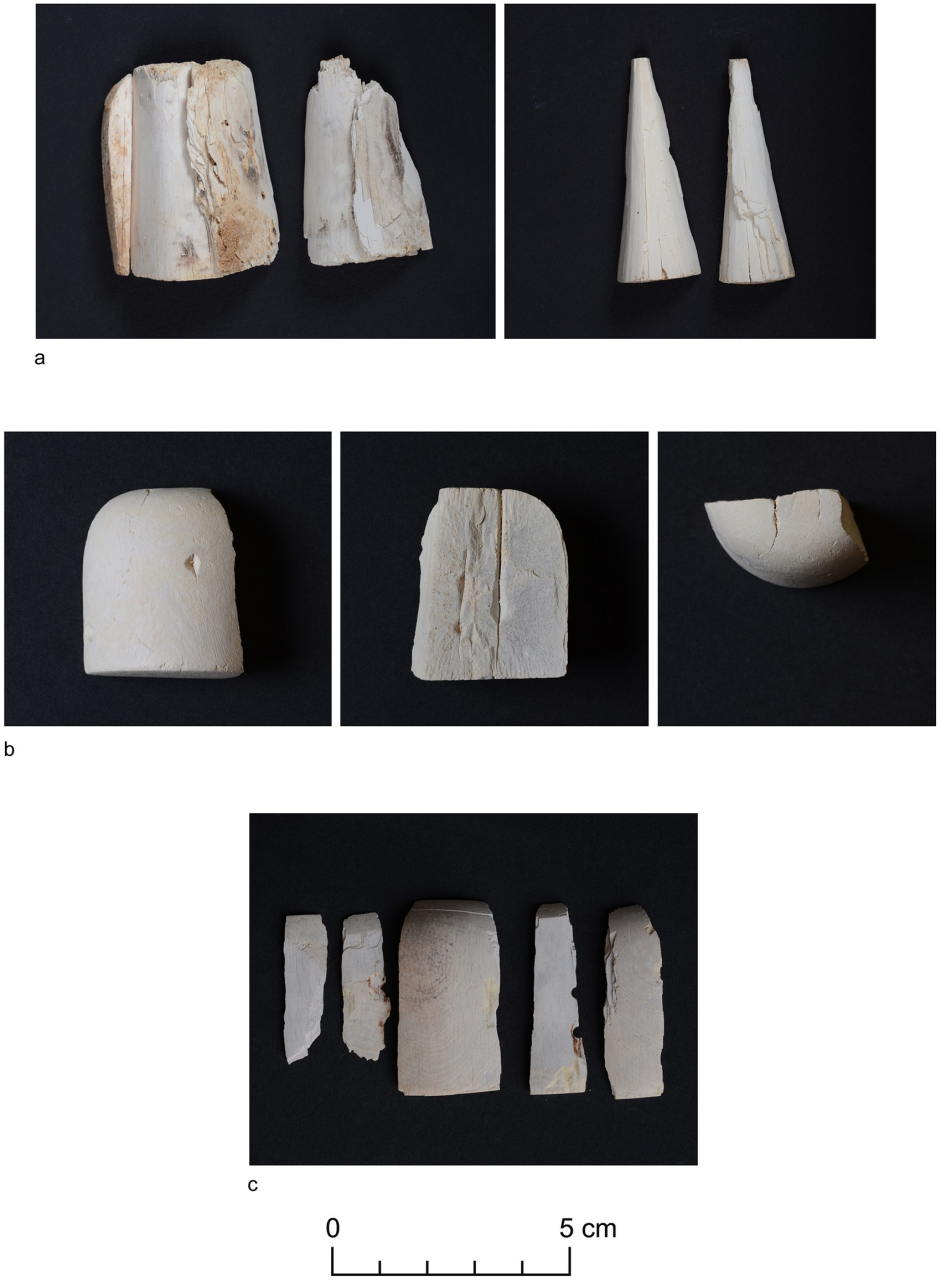
Fragments of ivory game pawns from tumulus 7 (Phot. Adam Kamrowski).

**Tumulus 7** is located in the northernmost edge of the tumuli field. The superstructure was about 8 m high with a diameter about 40 m. The two-branched tunnel of the tumulus was excavated in 2011 (sondage) and 2015 (Fig 4). It was 17 m long and contained fragments of weapons, archer’s looses, bed frame copper fittings, nails, copper plate with an incised cross, shell, stone and faience beads, fragments of glass, pieces of ivory game pawns (Fig 6) and animal bones. All the animal remains were found in one layer, some of them were left by the robbers in the vicinity of the entrance to the burial chamber (cervical vertebrae and radius which belonged to young cattle) (Table 4). The assemblage consists of 329 bone fragments, 92 fragments were unidentified. Only a few bones bore post-consumption marks, additionally, 3 bone fragments were burnt. It is characteristic that the bones of only two/three species—cattle and sheep/goat—were present in a greater number. The majority of animal remains came from cattle (216 fragments from only 33 bones and 1 tooth). Two individuals of different ages were represented in the assemblage: one about 5 years old, with a very robust skull and mandible and one under 15–20 months old. There was also one cervical vertebra from an animal aged more than 5 years. The bones came from the head of the older animal (skull, mandible, tooth), and other parts of both carcasses, mostly those bearing good quality meat: trunk (cervical, thoracic and lumbar vertebrae and ribs), proximal parts of the forelimb (scapula, humerus, radius) and hind limb (pelvis, femur, patella, tibia) from both sides of the carcass. Only a few bones came from distal parts of forelimb (metacarpal) and hind limb (ossa tarsi), there were no extremities.

**Table 4:** Animal bones from tumulus 7.

Species	Localization	Bone	Number of fragments (NISP)	Number of bones (MNE)			Description (MNI, age, additional information)
				Right	Left	Right/Left	
Cattle	Entrance to the burial chamber	Cervical vertebra	1			1	2 individuals: 1 about 5 years old, 1 under 15–20 months old
		Radius	1			1	
	Tunnel, upper layer in the entrance	Rib	7			1	
	Tunnel	Skull	1			1	
		Mandible	2			1	
		Tooth	1			1	
		Cervical vertebra	5			2	
		Thoracic vertebra	4			2	
		Lumbar vertebra	1			1	
		Rib	146	6	2		
		Scapula	5	1			
		Humerus	12	1	1		
		Radius	3	1			
		Metacarpal	6	1			
		Pelvis	1		1		
		Femur	12	1	1	1	
		Patella	1	1			
		Tibia	2		1	1	
		Talus	2	1	1		
		Ossa tarsi	2			2	
		Cervical vertebra	1			1	more than 5 years old
Sheep/goat	Tunnel, upper layer in the entrance	Rib	2			1	1 individual: about 3–5 month old
	Tunnel	Lumbar vertebra	4			1	
		Rib	10			3	
		Humerus	2		1		
		Radius	6			1	
		Pelvis	3	1		1	
Horse/donkey	Tunnel	Rib	1			1	adult
Dog	Tunnel	Tibia	2		1		adult

Only 27 fragments from 9 bones belonged to one sheep or goat about 3–5 month old.—The bones were broken into undiagnostic pieces. The bones came from parts of the carcass with good quality meat, such as trunk (lumbar vertebrae, ribs), proximal parts of the forelimb (humerus, radius) and hind limb (pelvis) from both sides of the carcass. Additionally, one rib was found close to the tunnel entrance (upper layer in the entrance).

There were only two bones that belonged to other animals than cattle and ovicaprids. A fragment of adult horse or donkey rib and two fragments of one left tibia of a dog were found in the tunnel. It is uncertain whether those bones were connected to the ritual or got to the fill of the tunnel accidentally as a result of burying it after the funeral or the robbery.

It seems that the bone material from tumulus 7 was very similar to the material from tumulus 3 and two other tumuli 6 and 8 as well. There were only two species that could be connected with the burial ritual, that is cattle (whose bones dominated in the material) and sheep (its presence was confirmed for the rest of the tumuli of type 1, there is no such certainty only in the case of tumulus 7 –the bones from tumulus 7 might have belonged to goat as well). In all the tunnels of tumuli of type 1 the animal bones were mixed together with human bones and artifacts, probably nothing remained *in situ* after looting. It is likely that the archaeological material that was found in the tunnels of tumuli of type 1 had partially been thrown away from the main chamber (or side chambers too), but it is also possible that part of it had been left there after some rituals that conducted in the tunnel.

**Tumulus 4** is located in the southern cluster of tumuli of type 1. Its diameter is about 34 m, its height– 6.2 m. Only the shaft leading to the tunnel and the tunnel itself were unearthed in seasons of archaeological works in 2013 and 2017 (Fig 4). The structure of the tunnel in the shape of two parallel branches with pillars between them was different from most of the other tumuli. Only tumuli 6 and 7 featured similar structures, all the other tunnels had only one corridor leading to the burial chamber. In the case of three tumuli mentioned above, one of the branches led to the burial chamber, the other was probably a blind corridor which ended with a rock wall, it is then unclear why such forms were hewn with a great effort in the rock. It appears that tumulus 4 was the most interesting tumulus at El-Zuma, equipped with the most numerous artifacts discovered at the site. Metal artifacts, such as arrowheads, nails, bed frame fittings and jewellery, such as a ring decorated with glass mosaic, silver pin, chain with two small crosses, bezels, bronze buckles, copper bell, together with pottery, human and animal bones were recovered from the tunnel fill. Most of the artifacts and some bones were found in a dark bottom layer in the western part of the tunnel.

3331 fragments of animal bones were discovered in both parts of the tunnel. They were in a very poor state of preservation, a lot of small fragments remained unidentified (more than 1/2 of the material– 1697 fragments). As in the case of the above-mentioned tumuli, cattle and ovicaprine bones dominated in the assemblage (Table 5).

**Table 5:** Animal bones from tumulus 4.

Species	Localization	Bone	Number of fragments (NISP)	Number of bones (MNE)			Description (MNI, age, additional information)
				Right	Left	Right/Left	
Cattle	Entrance to the room W	Thoracic vertebra	4			1	3 individuals: 1 female, about 3.5–4 years old; 1 male, more than 3–3.5 years old; 1 young individual, between 15 and 20 months old
		Femur	10	1			
		Talus	1		1		
	Tunnel part E	Skull	9			1	
		Mandible	105	1	3		
		Tooth	171		1	not less than 4	
		Cervical vertebra	1			1	
		Lumbar vertebra	5			3	
		Rib	45	2			
		Scapula	24	2	1		
		Humerus	16	2	2		
		Radius	18	2	1	1	
		Ulna	3	1	1		
		Metacarpal	11	1	1	1	
		Metacarpal or Metatarsal	1			1	
		Pelvis	4	1	1		
		Femur	13	1		2	
		Patella	1	1			
		Tibia	28	2	3		
		Calcaneus	3	1	2		
		Talus	3	1	2		
		Centroquartale	2			1	
		Ossa tarsi	1			1	
		Metatarsal	9	1		1	
	Tunnel part W	Skull	89			1	
		Mandible	7	1	1		
		Tooth	63		not less than 4		
		Lumbar vertebra	1			1	
		Sternum	6			3	
		Rib	56	2	not less than 4		
		Scapula	1			1	
		Humerus	3	1			
		Radius	5	1	1		
		Metacarpal	1	1			
		Metacarpal or Metatarsal	1			1	
		Pelvis	27	1	1		
		Femur	27	3	1		
		Tibia	29	3	2		
		Calcaneus	1		1		
		Talus	2	1	1		
		Ossa tarsi	1			1	
		Metatarsal	36	1	1	2	
		Ph I	3	1	1		
	Tunnel part W—dark bottom layer	Skull	1			1	
		Mandible	2			1	
		Tooth	5			1	
		Rib	27			2	
		Humerus	4	1			
		Radius	8			1	
		Pelvis	11	1			
		Femur	2		1		
		Tibia	17	1			
		Calcaneus	1	1			
		Talus	4	1	1		
Sheep	Tunnel part E	Skull	6			1	2 individuals: 1 under 5 months old; 1 between 5 months and 3–3.5 years old
		Mandible	20		1		
		Scapula	1		1		
		Radius	9	1			
		Femur	1		1		
		Talus	1		1		
	Tunnel part W	Femur	1		1		
		Talus	2	2			
	Tunnel part W—dark bottom layer	Scapula	9		1		
Goat	Tunnel part W	Skull	2			1	1 individual: between 2 and 3 years old
		Atlas	1			1	
		Humerus	1	1			
		Calcaneus	1	1			
		Metacarpal or Metatarsal	1			1	
Ovicaprine	Tunnel part E	Skull	22			1	probably from the sheep and goat above
		Tooth (Skull)	4		1		
		Tooth (Mandible)	1		1		
		Tooth	19			not less than 7	
		Sacrum	1			1	
		Rib	9			2	
		Scapula	4	1	1		
		Humerus	3		1		
		Radius	6		1		
		Ossa carpi	4			4	
		Femur	2		1		
		Tibia	7			2	
		Calcaneus	1			1	
		Ossa tarsi	1			1	
		Metatarsal	8	1		1	
		Ph I	1	1			
	Entrance to the room W	Cervical vertebra	1			1	
	Tunnel part W	Skull	5			1	
		Mandible	6		1	1	
		Tooth (Skull)	3			1	
		Tooth (Mandible)	10		1	2	
		Tooth	9			5	
		Cervical vertebra	1			1	
		Sternum	2			2	
		Rib	55	5	3	2	
		Scapula	6			1	
		Humerus	2	1		1	
		Radius	1		1		
		Metacarpal	1		1		
		Pelvis	2			1	
		Femur	1			1	
		Patella	1		1		
		Tibia	13	1		1	
		Calcaneus	1		1		
		Metatarsal	2			1	
	Tunnel part W—dark bottom layer	Mandible	4			1	
		Lumbar vertebra	2			1	
		Metacarpal	1			1	
Donkey	Tunnel part E	Humerus	2	1	1		1 individual: adult
		Metacarpal	2			1	
		Femur	5	1	1		
		Tibia	3		1		
		Ph I	1		1		
	Entrance to the room W	Rib	5	1			
		Pelvis	17	1			
	Tunnel part W	Radius	2		1		
		Ulna	1	1			
		Ossa carpi	1		1		
Camel	Tunnel part E	Skull	8			1	2 individuals: 1 almost adult; 1 adult
		Mandible	3	2	1		
		Thoracic vertebra	1			1	
		Lumbar vertebra	1			1	
		Rib	20	3			
		Scapula	2	1	1		
		Humerus	3		1		
		Femur	1	1			
		Metatarsal	4		1		
	Tunnel part W	Mandible	4			1	
		Scapula	16		1		
		Centroquartale	2	1			
		Calcaneus	1	1			
		Metatarsal	5	1			
Gazelle	Tunnel part E	Humerus	8		1		adult
Gazella dorcas	Tunnel part W	Skull	1				female
Carnivore	Tunnel part E	Lumbar vertebra	1			1	adult
Carnivore	Tunnel part W	Rib	2			2	1 individual
Dog	Tunnel part E	Lumbar vertebra	2			1	young
Bat	Tunnel part E, top of the filling	Skull	14			5	5 individuals: adult
		Mandible	15	4	3		
		Atlas	2			2	
		Cervical vertebra	10			10	
		Thoracic vertebra	2			2	
		Lumbar vertebra	4			4	
		Rib	28			28	
		Clavicle	1		1		
		Scapula	3	1	2		
		Humerus	6	3	3		
		Radius	3	1	1		
		Ulna	8	3	2	1	
		Metacarpal	1			1	
		Pelvis	3			3	
		Femur	2	1			
		Tibia	3	2	1		
		Metatarsal	2			2	
		Fragments of long bones	15			15	
	Tunnel part E	Skull	2			2	3 individuals: adult
		Mandible	4	2	2		
		Atlas	2			2	
		Axis	2			2	
		Cervical vertebra	8			8	
		Thoracic vertebra	4			4	
		Lumbar vertebra	4			4	
		Sternum	2			2	
		Clavicle	1			1	
		Rib	26			26	
		Scapula	4	2	2		
		Humerus	4	2	2		
		Radius	2			1	
		Ulna	6	2	3		
		Metacarpal	10			8	
		Pelvis	2	1	1		
		Femur	5	2	2		
		Metatarsal	1			1	
		Ph I	1			1	
		Fragments of long bones	10			10	
	Tunnel part W, top of the filling	Skull	4			1	1 individual: adult
		Mandible	4	1	1		
		Cervical vertebra	4			4	
		Thoracic vertebra	3			3	
		Sacrum	1			1	
		Sternum	1			1	
		Rib	12			12	
		Scapula	4	1	1		
		Humerus	2	1	1		
		Ulna	3	1	1		
		Metacarpal	2			2	
		Pelvis	2			1	
		Tibia	2	1	1		
		Ph I	1			1	
		Fragments of long bones	27			27	
	Tunnel part W	Skull	2			1	2 individuals: adult
		Mandible	1		1		
		Humerus	3	1	2		
		Ulna	3	1	1		

The most numerous group of bones belonged to cattle (929 fragments from 124 bones). The remains came from three animals: one male older than 3–3.5 years, one young individual between 15 and 20 months old and one female about 3.5–4 years old, with withers height of about 120.4–124.7 cm. Withers height was calculated on the basis of a tibia (GL-349 mm) and metatarsal (GL-233 mm) (Table 1). It seems that the fragments from the head (skull, mandible, teeth) came from the male individual, as well as some long bones with the thicker compact bone than in other specimens. On the basis of those elements, however, it was impossible to establish the height at the withers. Bones from the trunk (cervical, thoracic and lumbar vertebrae, sternum, ribs), proximal parts of the forelimb (scapula, humerus, radius, ulna) and hind limb (pelvis, femur, patella, tibia) belonged to all three animals, as well as the bones from distal parts of the forelimb (metacarpal) and hind limb (ossa tarsi, metatarsal). It is also interesting that two phalanges from one of the two adult animals were also found in the material from the tunnel. A few cattle bones were discovered in the vicinity of the entrance to the western room that was not excavated because of the collapse of the ceiling.

Ovicaprine bones were less numerous. 278 belonged to these animals, but in the case of 222 it was impossible to distinguish the species. The fragments came from different parts of the body, such as the head (skull, mandible, mandibular and skull teeth), trunk (cervical and lumbar vertebra, sacrum, sternum and ribs) and proximal and distal parts of the forelimb (scapula, humerus, radius, ossa carpi, metacarpal) and hind limb (pelvis, femur, patella, tibia, calcaneus, ossa tarsi, metatarsal). Additionally, one right proximal phalanx was discovered in the eastern part of the tunnel. 50 fragments from 10 sheep bones were found in all the parts of the tunnel, however only one left scapula came from the dark bottom layer located in the western part of tunnel. They represented the head (skull, mandible) and proximal parts of the forelimb (scapula, radius) and hind limb (femur). Apart from that, 2 sets of talus were also found. The bones belonged to two animals of different ages. One of them was very young, under 5 months old, the other was bigger and older, aged between 5 months and 3–3.5 years old. Goat bones were very rare, only 6 fragments from 5 bones from different parts of the skeleton were found in the eastern part of the tunnel (skull, atlas, humerus, calcaneus, metacarpal or metatarsal). All of them probably belonged to one individual between 2 and 3 years old. All the other ovicaprine bones probably came from the sheep and goat as mentioned above.

Except for the remains of these three species, present in other tumuli of type 1, in tumulus 4 there were also bones that belonged to transportation animals, such as camel and donkey. 71 fragments from 19 camel bones were recovered from both parts of the tunnel. The skeletons of two individuals, one almost adult and one adult were incomplete, only the head (skull, mandible), trunk (thoracic vertebrae, lumbar vertebra, ribs), proximal parts of the forelimb (scapula, humerus) and hind limb (femur) and distal part of the hind limb (centroquartale, calcaneus and metatarsal) were discovered. In the case of donkey, the bones were also scattered in both parts of the tunnel, additionally one rib and pelvis were found in the entrance to room W. All the bones probably belonged to one adult animal and came from all the parts of the skeleton but the head (rib, humerus, radius, ulna, ossa carpi, metacarpal, pelvis, femur, tibia, phalanx I).

A partially preserved skull of a female gazelle (Fig 5) and one humerus in 8 pieces were discovered in both parts of the tunnel. These elements probably came from one individual of *Gazella dorcas*. There were also fragments of 4 carnivore bones: 2 lumbar vertebrae (one of them clearly belonged to a dog) and two ribs. A dog vertebra came from a young animal while other bones belonged to an adult individual.

Among the animal bones excavated in the tunnel there were also 11 skeletons of adult bats (303 fragments from 266 bones). Some of the skeletons were incomplete (Fig 5). Five of them were found on the top of the fill of the eastern part of the tunnel, additionally—one was discovered in the top of the fill of the western part of the tunnel. Another 3 skeletons were located in lower layers of the eastern part of the tunnel and two more in the western part of the tunnel. The presence of these animals is easy to explain if it is assumed that the tunnel remained open either after the funeral or after the robbery. It seems that both situations are likely, as bat bones were found in top layers and lower parts of the tunnel fill as well.

Tumulus 4 was certainly exceptional. The discovery of the remains of transportation and game animals shows its unique character. The only similar grave offerings were found in tumulus 1 of type 1. It is uncertain whether the whole animals were deposited in the grave (tunnel or the western chamber or both of them). The contents of the tunnel were mixed up, probably during the robbery, bones which belonged to one animal were discovered in both corridors of the tunnel on different levels. However, it is certain that the skeletons of the transportation animals were incomplete: in the case of two skeletons of camels, phalanges were the missing elements, in the case of the donkey, the head of the animal was not found. The remains of cattle and sheep also came from incomplete skeletons. On the other hand, only the tunnel was explored, the rest of the tumulus still remains unexplored. Additionally, all the bones were in a very poor state of preservation, they were broken into many pieces, sometimes even almost crushed into powder, which might have caused the disappearance of some bone elements, but even then, teeth fragments and some elements from the skull should have remained on the spot, as it was in the case of tumulus 1.

#### Tumuli of type 2

Tumuli of type 2 are the best represented tumuli at El-Zuma. There are 13 features of this kind, mostly in the central part of the tumuli field: numbers from 9 to 16, from 23 to 26 and number 29 (Fig 3). The latter one has not been excavated so far, apart from that, tumulus 12 was unearthed only partially in two excavation seasons. All the other tumuli were explored completely: shafts and chambers were excavated after removing the superstructures. Bone assemblages from tumuli 10 and 25 of type 2 excavated in season 2007 were described by M. Osypińska [6].

The ceilings in the main chambers of most of the tumuli of type 2 collapsed. In most of the cases it was caused by robbers’ activity, only in one case it might have happened as a result of poor quality of the rock in which the chamber was cut and some natural processes (tumulus 26). In tumulus 13 the ceiling of the main chamber collapsed during the excavations. The side chambers were usually untouched but in tumuli 9, 14, 15, 24 and 26 chamber 2, next to the main chambers, the fragments of the collapsed ceilings covered the floor which was probably caused by robbers’ activity.

The typical bone offerings deposited in graves of type 2 will be presented on the example of the assemblage from tumulus 14. In all the tumuli of type 2 but one the southern chamber functioned as the main burial chamber. The localization of the burial chamber in the peripheral tumulus 12 is unclear, as the southern chamber did not contain a human body, however after two seasons the excavations had to be stopped because of the danger of collapse of the sandstone structure of the grave, so the contents of chamber 2 remain unknown.

Bones from following tumuli were selected for aDNA analysis: tumulus 11 (scapula, humerus, femur), tumulus 12 (humerus, radius, femur, tibia), tumulus 13 (humerus, pelvis, femur), tumulus 14 (2 scapulas, 4 humeri, femur, tibia), tumulus 15 (scapula, humerus), tumulus 16 (humerus, tibia), tumulus 24 (humerus, femur, tibia), tumulus 25 (humerus), and tumulus 26 (humerus, pelvis, tibia).

**Fig 7:**
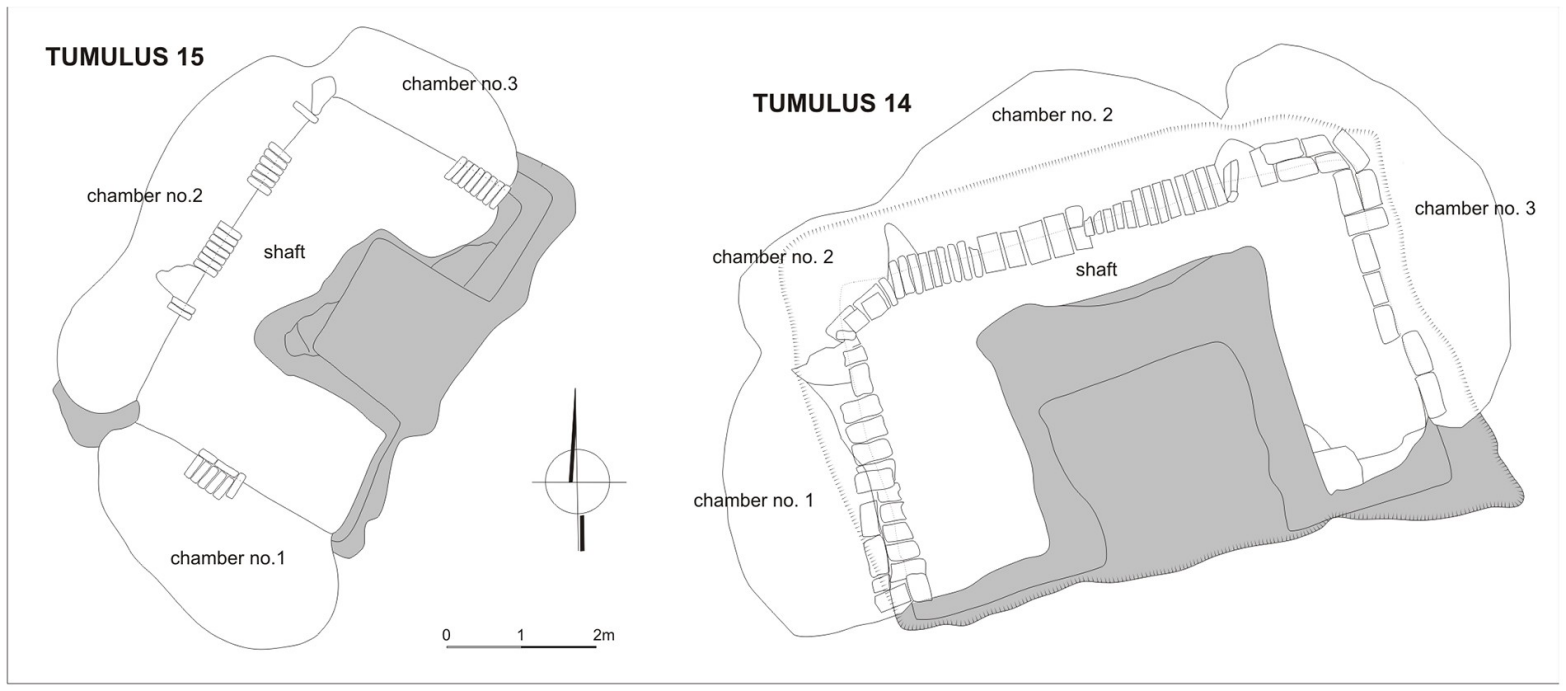
El-Zuma. Plans of tumuli of type 2: tumulus 14 (draw. K. Solarska, E. Skowrońska, J. Brochocki, digit. K. Solarska, J. Brochocki, E.Czyżewska) and tumulus 15 (draw. E. Skowrońska, digit. E.Czyżewska).

**Tumulus 14** is located in the northern part of the central cluster of tumuli of type 2 and is one of the biggest objects of this kind. The superstructure was 2.79 m high, the shape was slightly irregular with a diameter on the WE axis of about 32 m. The exploration of the U-shaped shaft and the three chambers of the tumuli (Fig 7) took place in season 2012. The animal offerings were present in all the three chambers. In the burial chamber the animal remains were mixed together with human bones, pottery and some metal nails and distributed in the whole area, most of them, however, were located in a cluster in the central part of the chamber. The side chambers contained only animal bones and pottery *in situ*. In chamber 2 the animal remains were concentrated in the central part, in chamber 3 they were distributed in four clusters.

The total number of animal bones from tumulus 14 was 538 fragments, 68 small fragments remained unidentified. Bones were in a slightly better state of preservation than the remains from tumuli of type 1, though they were dry and preserved in pieces.

Cattle remains (278 fragments from 29 bones) were found in all three chambers (Table 6). They came from the trunk (cervical vertebrae, thoracic vertebrae, sternum, ribs), proximal parts of the forelimb (scapula, humerus) and hind limb (femur, patella, tibia), additionally some bones from the distal part of the hind limb (talus, ossa tarsi) were excavated in chamber 3. They represented two individuals: one between 2–2.5 and 3.5–4 years old and one slightly older between 3.5–4 and 5 years old.

**Table 6:** Animal bones from tumulus 14.

Species	Localization	Bone	Number of fragments (NISP)	Number of bones (MNE)			Description (MNI, age, additional information)
				Right	Left	Right/Left	
Cattle	chamber 1	Cervical vertebra	1			1	2 individuals: 1 between 2–2.5 and 3.5–4 years old; 1 between 3.5–4 and 5 years old
		Thoracic vertebra	1			1	
		Sternum	1			1	
		Rib	43	1	1	1	
		Scapula	1	1			
		Femur	16		1		
		Patella	1			1	
	chamber 2	Rib	20			not less than 4	
		Humerus	6	1			
	chamber 3	Sternum	6			3	
		Rib	94	1		not less than 6	
		Scapula	78		1		
		Humerus	6	1			
		Tibia	2	1			
		Talus	1	1			
		Ossa tarsi	1			1	
Sheep	chamber 2	Humerus	5		1		2 individuals: 1 under 3.5 years old; 1 about 3.5 years old
		Radius	1		1		
		Ulna	3		1		
		Tibia	17	1	1		
		Calcaneus	3		2		
		Talus	2	1	1		
Ovicaprine	chamber 1	Humerus	1			1	probably from 2 sheep above
		Tibia	2			1	
	chamber 2	Rib	13			not less than 2	
		Scapula	3			1	
		Ossa carpi	1			1	
		Pelvis	6	1			
		Ossa tarsi	1			1	
		Centroquartale	1	1			
	chamber 3	Rib	110	1	1	not less than 6	
		Sternum	11			not less than 2	
		Pelvis	5	1			
		Femur	6	1	1		
		Patella	1			1	

Ovicaprine remains were equally frequent: 31 fragments from 9 bones belonged to sheep and another 161 fragments from 23 bones could not be identified to the level of species, but it is likely that the bones came from two sheep, whose bones could be identified. Sheep bones were found in chamber 2 and came from the proximal part of the forelimb (humerus, radius, ulna) and proximal and distal parts of the hind limb (tibia, calcaneus, talus) and they belonged to two 2 individuals: one under 3.5 years old and one about 3.5 years old. The height at the withers of the latter animal was between 61.4 and 67.6 cm, approximately 65.4 cm. Withers height was calculated on the basis of a femur (GL-174 mm) and two talus bones (GLl-29.6 and 30 mm) (Table 1). Unidentified ovicaprine bones found in all three chambers of the tumulus came from the trunk (rib, sternum), proximal and distal parts of the forelimb (scapula, humerus, ossa carpi) and hind limb (pelvis, femur, patella, tibia, ossa tarsi, centroquartale).

It seems that the animal offerings deposited in tumulus 14 were typical for this type of graves. Similar animal remains that came only from cattle and ovicaprids (most probably sheep only, as the goat remains were not found in grave contexts) are known from other tumuli of type 2 at El-Zuma, such as tumuli 11, 12, 13, 15, 16, 24 and 26. Only tumulus 9 differed as to the bone contents—only a few ovicaprine bones were found in the course exploration but the grave was damaged by collapsing ceilings and the fill of the chambers was very wet which might have caused the destruction of the botanical and zoological remains.

#### Tumuli of type 3

The tumuli of type 3 (numbers 17–22 and 27–28) were the smallest features at El-Zuma as to the super- and substructures. They were concentrated in the southern part of the concentration of tumuli of type 2 in the central part of the tumuli field (Fig 3). All the tumuli contained only a shaft and a burial chamber cut in the western or north-western wall of the shaft. All of them had been looted. The state of preservation of the tumuli of type 3 was quite good. In most of the cases the robberies did not destroy the constructions of the chambers. There were but three exceptions: the ceilings of the chambers in tumuli 17 and 21 collapsed because of robbers’ activity while in chamber 20 probably because of poor quality of the rock in which the chamber was cut.

It is interesting that two of tumuli of type 3 (tumuli 18 and 27) did not contain animal remains at all. One of the most typical tumuli of this kind as to the animal offerings was tumulus 20. Two tumuli (17 and 19) contained slightly different offerings, that is why the contents of one of them, tumulus 17, will also be discussed below.

No samples were taken for aDNA research from these structures. The few cattle bones found in tumuli of type 3 were in a bad state of preservation and they were not suitable for such an analysis.

**Fig 8:**
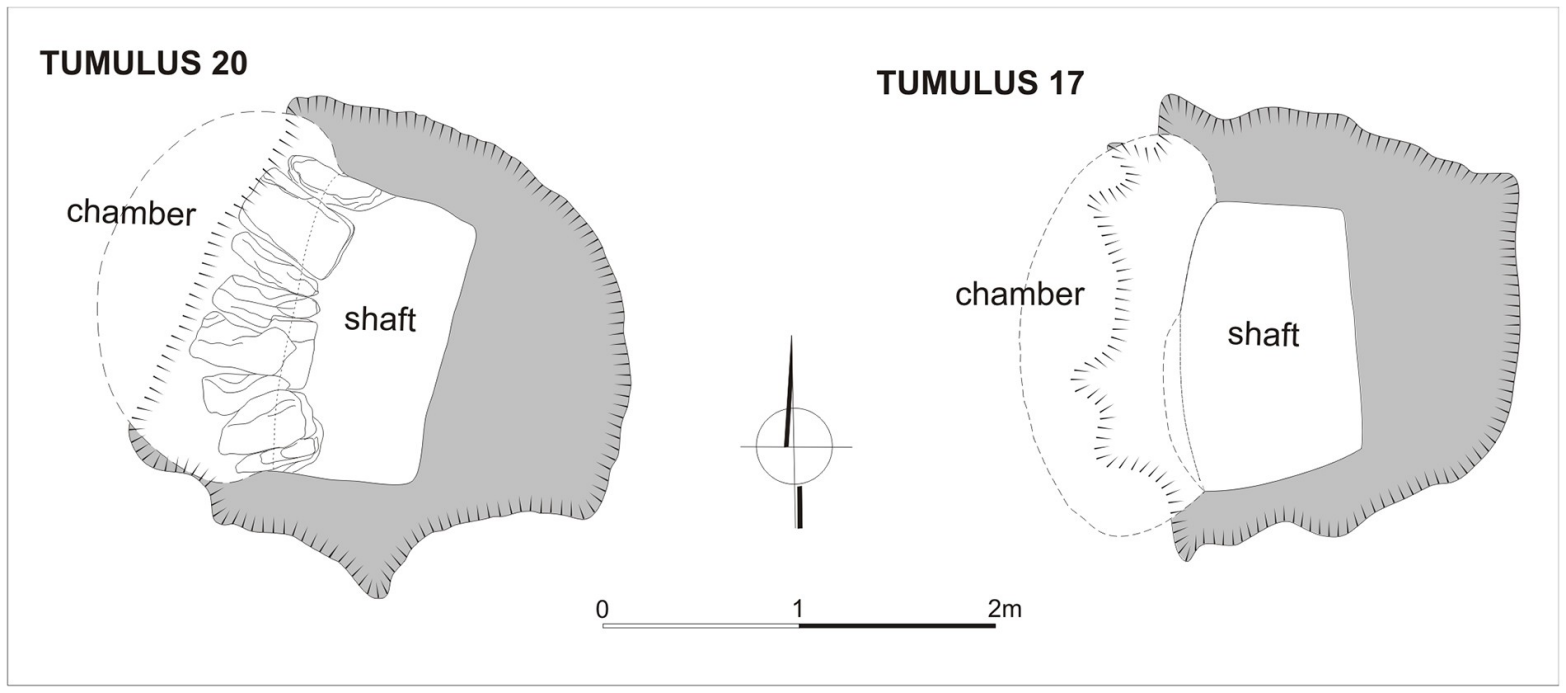
El-Zuma. Plans of tumuli of type 3: tumulus 20 (draw. K. Juszczyk, E. Czyzewska, E. Klimaszewska-Drabot, digit. E.Czyżewska), tumulus 17 (draw. E. Czyżewska, E. Klimaszewska-Drabot, digit. E.Czyżewska).

**Tumulus 20** is one of the smaller tumuli at El-Zuma. It was only 70 cm high with a diameter of about 13.5 m. The shaft and the burial chamber were excavated in season 2011 (Fig 8). It contained very similar offerings as tumulus 21, the best equipped tumulus of type 3 [7]. The human and animal remains were distributed in the shaft and the chamber together with pottery (bottles and cup) and beads. It seems that a human body and some other offerings were dragged away from the chamber during the robbery.

There were only 13 bone fragments in the tumulus. The animal bones were present mostly in the shaft, only two fragments of one pelvis were found in the chamber. In the burial context there were only ovicaprine remains (13 fragments from 7 bones from the head and limbs) (Table 7). Two of the ovicaprine bones could be identified to the species—a humerus and talus belonged to one sheep aged under 3–3.5 years old. There were no goat remains in this tumulus, it seems likely therefore that only sheep bones from the mandible and both proximal parts of the forelimb and hind limb were found in the tomb.

**Table 7:** Animal bones from tumulus 20.

Species	Localization	Bone	Number of fragments (NISP)	Number of bones (MNE)			Description (MNI, age, additional information)
				Right	Left	Right/Left	
Sheep	shaft (between human bones)	Humerus	1			1	1 individual: under 3–3.5 years old
		Talus	1			1	
Ovicaprine	chamber	Pelvis	2		1		probably from sheep above
	shaft (between human bones)	Mandible	4			1	
		Scapula	2			1	
		Ulna	1			1	
		Pelvis	2	1			

**Tumulus 17** was also one of two smallest features at the site. Its diameter was about 10 m, it was barely visible on the surface with its height of about 45 cm. The shaft and the burial chamber were excavated in season 2009 (Fig 8): it contained pottery, arrowheads, beads and an iron ring. Only 20 bone fragments were discovered in the burial chamber (Table 8), 5 of them remained unidentified. Most of the bones (8 fragments from 5 bones) belonged to one sheep aged between 3 and 3.5 years old. They came from the right hind limb (femur, tibia, talus, calcaneus, centroquartale). Some other remains (two pieces of sternum, rib, sesamoid) belonged to ovicaprids, probably the same individual of sheep as the rest of the bones. It was also interesting that among the ovicaprine bones 3 fragments of one cattle ulna were also found. Such a situation was observed only in tumulus 19. In the other tumuli of type 3 analyzed by the first author only sheep bones were discovered.

**Table 8:** Animal bones from tumulus 17.

Species	Localization	Bone	Number of fragments (NISP)	Number of bones (MNE)			Description (MNI, age, additional information)
				Right	Left	Right/Left	
Cattle	Chamber	Ulna	3			1	1 individual
Sheep	Chamber	Femur	4	1			1 individual: between 3 and 3.5 years old
		Tibia	1	1			
		Talus	1	1			
		Calcaneus	1	1			
		Centroquartale	1	1			
Ovicaprine	Chamber	Sternum	2			2	probably from sheep above
		Rib	1			1	
		Sesamoid	1	1			

It seems that in the case of tumuli 17 and 20, as well as in the case of other tumuli of type 2 and 3 only food offerings were deposited. There were no head fragments and no extremities, mostly parts of the carcass with good quality meat, such as the trunk and proximal parts of the forelimb and hind limb were used during burial rituals. Probably, when bigger pieces of the carcass were involved, distal parts but without extremities were also added. A set of the tumuli of type 3 (tumulus 20, 21, 22 and 28) were equipped exclusively with pieces of sheep carcasses, partially in a form of bigger cuts of meat (it is uncertain if they were prepared in any way), partially in a form of dishes served in bowls, e.g. in tumulus 21 [7]. There were, however, two exceptions from this rule: tumulus 17 and 19 which contained also a cattle and sheep offerings.

### El-DETTI tumuli field

**Fig 9:**
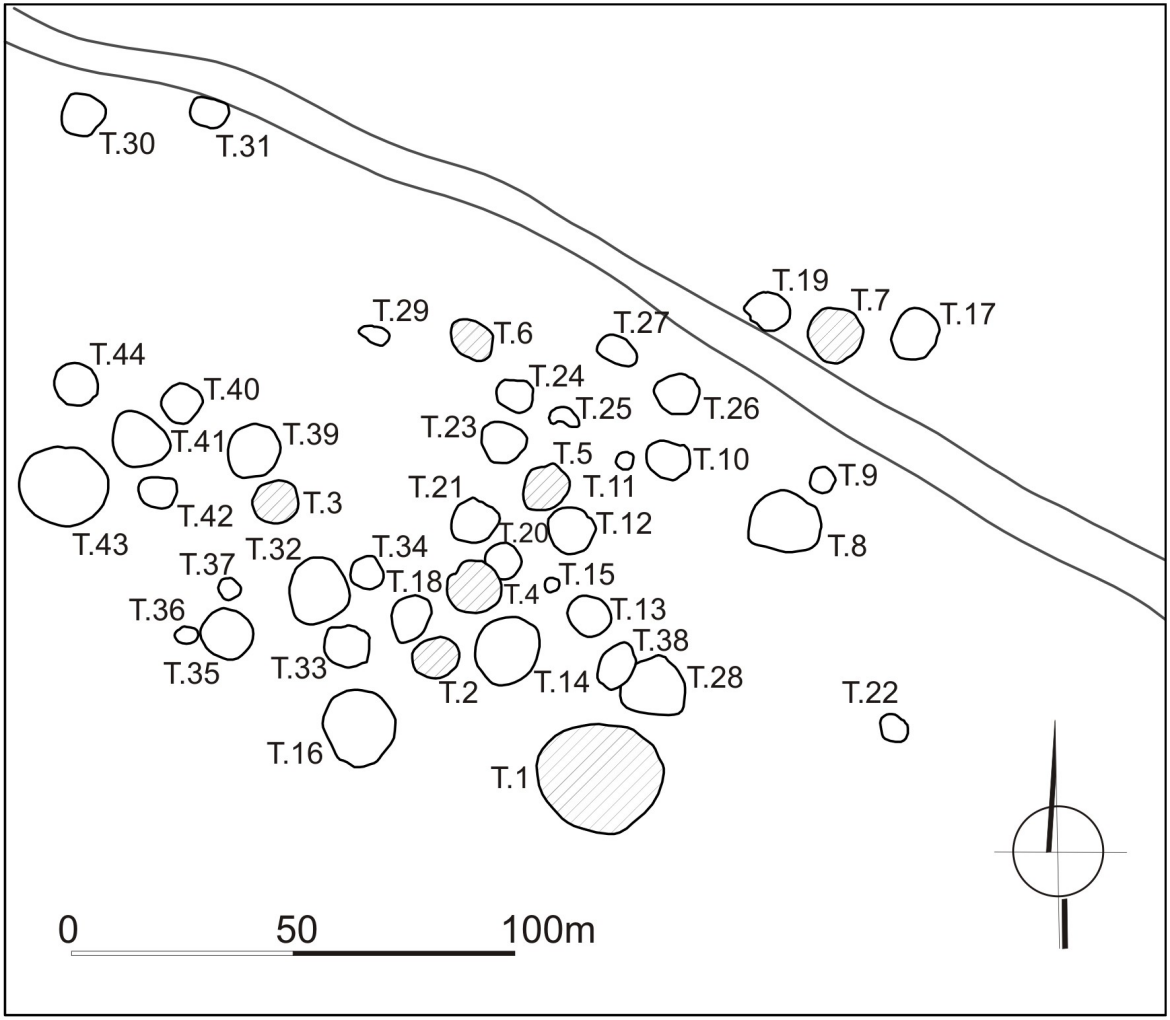
Plan of the El-Detti tumuli field (Copyright Makuria Research Project, Polish Centre of Mediterranean Archaeology of the University of Warsaw).

The tumuli field at El-Detti is located only 7 km up the river from El-Zuma village. Only 7 tumuli (tumuli 1–7) were excavated out of 44 features of this kind known from this site (Fig 9) [36]. All the explored graves were relatively small, most of them (tumuli 2–6) were of type 3. Only tumulus 1 could be considered as a tumulus of type 2 according to the classification by A. Obłuski [35] and El-Tayeb [13]. The tumuli field was dated to the 2nd phase of the Early Makurian period. All the excavated graves had been looted, just as it was in the case of the El-Zuma tumuli field. Animal remains, together with other artifacts, were discovered on the chambers floor and in the fill of the shafts. The offerings situated in the chambers of tumuli 2, 3, 4, 5 and 7 were covered by the fragments of the sandstone ceiling. The chamber of the tumulus 4 and the main chamber (chamber 1) of tumulus 1 were looted, the blockages were damaged and the fill of the shaft covered almost whole chambers. The other chambers (chamber 2 and 3) in tumulus 1 were intact. Because of the collapsing of the ceilings part of the animal remains was fragmented.

The animal remains from all the excavated tumuli from El-Detti were analyzed [8], however, only the ritual context of the animal offerings was discussed, that is why a short description of the tumuli contents (tumuli 1, 2, 4 and 7) is provided below and animal offerings will be discussed in a quite different economic aspect.

#### Tumuli of type 2

The only representative of tumuli of type 2 excavated by of Mahmoud El-Tayeb’s team was tumulus 1. It was at the same time the biggest grave in the El-Detti tumuli field. In many aspects the contents of this tumulus was similar to such objects at El-Zuma.

Tumulus 1 was the biggest object at the site with a diameter of about 25–28.5 m and height of the superstructure of about 1.7 m. It was located in the southern part of the tumuli field. The underground structures of the tumulus, such as its L-shaped shaft and three chambers, were excavated in 2015. Only the main burial chamber (chamber 1) bore traces of looting, two other chambers seemed to be untouched. Pottery (bottles, bowls, cups) and blue faience beads were found in all underground structures. In chamber 1 fragments of burned wood were also discovered. Human and animal bones mixed together were located in three clusters in chamber 1, animal bones were also recovered from room 2.

Though the state of preservation of the animal remains was generally poor, only 7 fragments out of 117 were unidentified. Animal bones were dry, some of them were also weathered. Cattle remains (61 fragments from 14 bones) were found in the shaft and chambers 1 and 2. They came from one individual aged about 3.5–4 years old. The remains came from the trunk and both the forelimb and the hind limb (almost exclusively from proximal parts), from the left and right sides of the carcass. Sheep bones (56 fragments from 20 bones) were also discovered in the shaft and chamber 1, additionally one fragment of tibia was found in chamber 2. The remains belonged to one individual under 3–3.5 years old. The animal was lame (a broken trochanter of the left humerus was not entirely healed). As it was in the case of cattle remains, sheep bones came from the trunk, the forelimb and the hind limb (mostly from proximal parts), from both sides of the carcass.

Three cattle samples (humerus and 2 tibias) were collected from tumulus 1 for aDNA analysis.

#### Tumuli of type 3

The group of 6 excavated tumuli of type 3 (tumuli 2–6) consisted of tombs (with a shaft and the only burial chamber) located in the central part of the site. They were of medium size in comparison with other structures from this site. The diameter of most of them (except for tumulus 7) was between 9.55 and 12.30 m, the height of the superstructure was 0.35–0.7 m. Tumuli 3, 5 and 6 contained typical graves with similar animal offerings and very poor equipment: mostly pottery, but in tumuli 5 and 6 there were also beads. Additionally, a few arrowheads were found in the shaft and the burial chamber in tumulus 5. The animal remains came exclusively from sheep carcasses. All the bones came from the trunk and limbs.

Tumulus 7 was separated from that group by the currently used road. It was one of the most east located tumuli with the size slightly bigger than the rest of excavated graves of type 3 (diameter was 12.3 m, its height was about 85 cm). It was also one of the most astonishing features as to the food offerings. It differed from the other tumuli of type 3 as well as from tumuli of type 3 known from El-Zuma site. It contained numerous fragments of pottery, beads and some metal objects. Among animal offerings there were only 6 sheep or goat ribs and an assemblage of cattle remains of a very young individual under 7–10 months old (ribs and bones from the proximal part of the left hind limb). A fragment of the right tibia was also found in the shaft and it came from a different individual then the rest of the bones. It might have been unconnected with the ritual offerings and got there accidentally when the robbery trench was filled with the sand from the desert due to some natural processes. It was the first known case when cattle remains dominated in the assemblage from tumulus of type 3. Two samples for aDNA analysis of cattle remains were taken from this tumulus (pelvis, tibia).

There were also two other tumuli (tumuli 2 and 4) whose contents differed from typical tumuli of type 3 at El-Zuma and El-Detti sites. In both of them pottery, some metal objects, beads (in tumulus 4 there was also a bone pendant of ‘an Egyptian Bes’ shape) and parts of sheep carcasses bearing good quality meat, typical for tumuli of type 3 food offerings, were unearthed, some of them bearing post consumption marks (Fig 2). It is worth noting that it was possible to determine the height at the withers (67 cm) for a sheep from tumulus 4. Tumuli 2 and 4 were exceptional, however, because they also contained dog remains which were with high probability connected with some burial rituals. Such dog burials in human graves are known from other Early Makurian sites, though they were rather rare finds (see discussion in [8], pages 444–445). Nevertheless, they were not found in the graves in the El-Zuma tumuli field. The remains of dogs (in each grave the remains of only one dog were discovered) were deposited either under the slabs that constituted the blockage or in the shaft, just near the wall of the blockage where the highest number was found. In the latter case, falling slabs (e.g. during the grave robbery) could have covered the dog remains. Unfortunately only parts of dog carcasses were preserved and additionally only in the case of the dog from tumulus 2, part of the body was articulated, so it is impossible to discuss whether the dogs were placed in the graves alive or after killing.

### Phylogenetic analysis of two mitochondrial DNA sequences obtained from bones collected at El Detti and El Zuma sites

We were able to isolate DNA from nearly all processed samples although out of 55 analyzed bones and teeth, only 2 yielded replicable and reliable mitochondrial d-loop sequences—one from El-Zuma site, no. ZS39 (length 273 bp) and another from El-Detti site, no. DS3 (length 169 bp). Poor amplification and sequencing results were caused by extremely poor quality of obtained samples. Bones were extremely dry and brittle. Obtained sequences have been deposited in GenBank (ZS39—MG892465; DS3—MG892466). None of the blank controls returned positive results.

Mitochondrial DNA (mtDNA) is widely used in phylogenetic studies due to its unique features—it is only maternally inherited, exists in the cell in multiple copies and undergoes rapid changes due to exposure to reactive oxygen species and poor DNA repair mechanisms [37]. Mutation rate of mitochondrial DNA is 10–17 fold higher that in genomic DNA thus mtDNA analysis reflects phylogenetic status of closely related species or even breeds (extensively reviewed by Gupta et. al. [38]). Sequence lengths obtained from ancient samples usually vary from 150 to 500 bp, which is usually sufficient to deliver reliable results for analysis [39].

**Fig 10:**
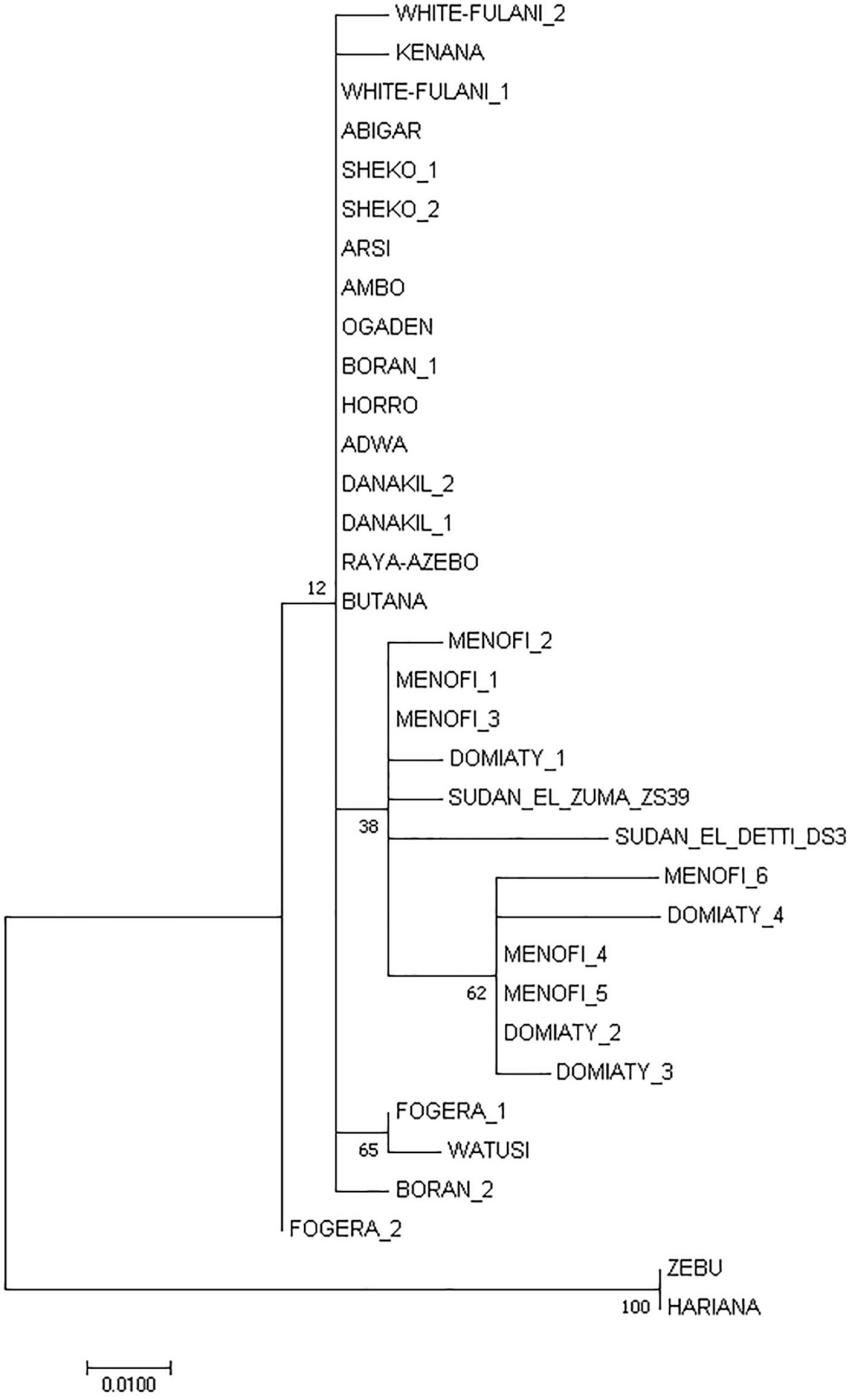
Molecular phylogenetic analysis by Maximum Likelihood method. The evolutionary history was inferred by using the Maximum Likelihood method based on the Tamura-Nei model [29]. The tree with the highest log likelihood (-406.58) is shown. The percentage of trees in which the associated taxa clustered together is shown next to the branches. Initial tree(s) for the heuristic search were obtained automatically by applying Neighbor-Join and BioNJ algorithms to a matrix of pairwise distances estimated using the Maximum Composite Likelihood (MCL) approach, and then selecting the topology with superior log likelihood value. The tree is drawn to scale, with branch lengths measured in the number of substitutions per site. The analysis involved 34 nucleotide sequences. Codon positions included were 1st+2nd+3rd+Noncoding. All positions containing gaps and missing data were eliminated. There were a total of 158 positions in the final dataset. Evolutionary analyses were conducted in MEGA7 [27].

**Fig 11:**
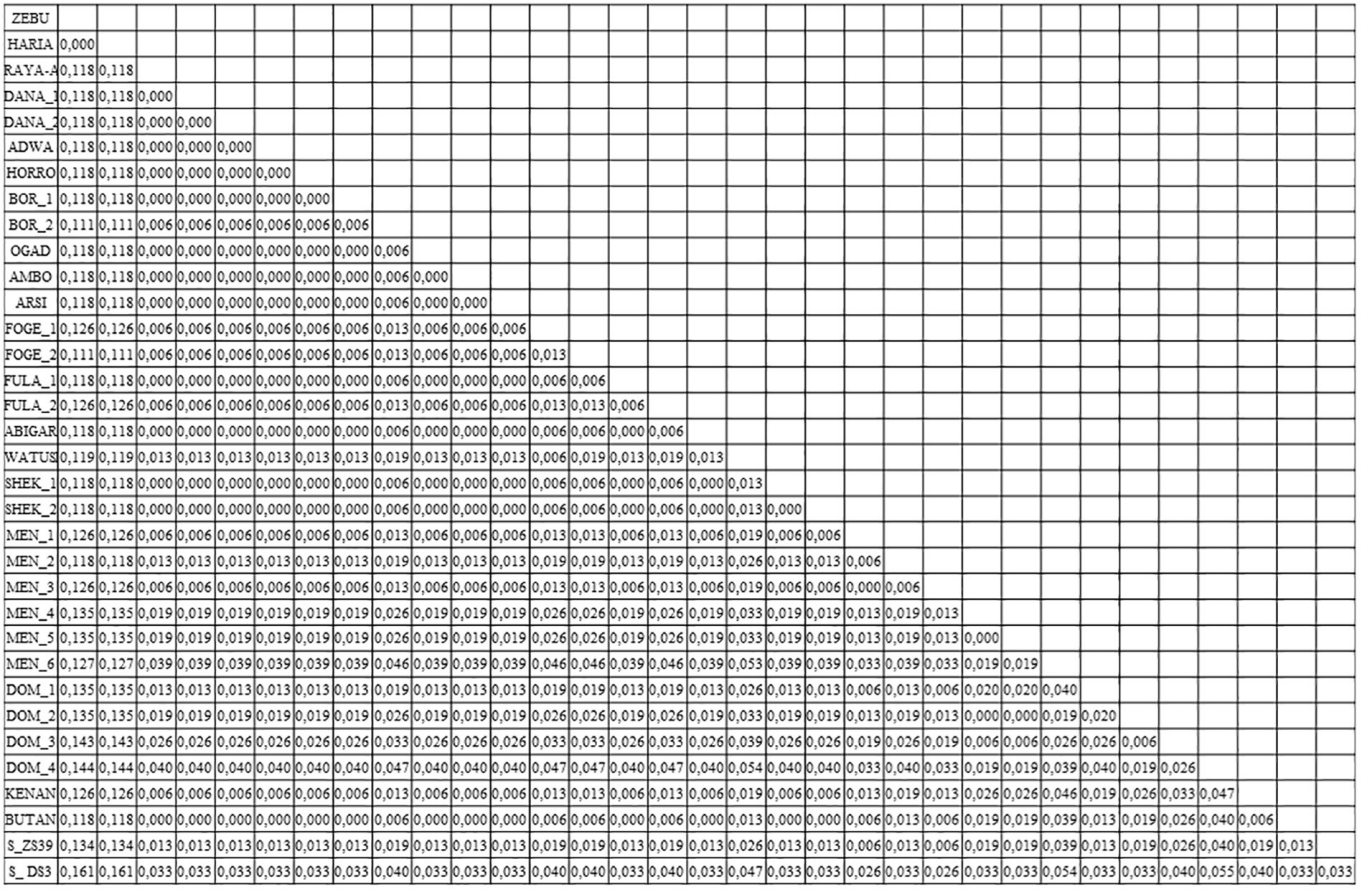
Estimates of evolutionary divergence between sequences. The number of base substitutions per site from between sequences are shown. Analyses were conducted using the Maximum Composite Likelihood model [28]. The analysis involved 34 nucleotide sequences. Codon positions included were 1st+2nd+3rd+Noncoding. All positions containing gaps and missing data were eliminated. There were a total of 158 positions in the final dataset. Evolutionary analyses were conducted in MEGA7 [27].

Although sequences obtained from ZS39 and DS3 were too short to provide reliable information about the haplogroup, according to Dittmar et. al. [39] such data can be a basis for a good description of phylogenetic relationship. To determine the phylogenetic relationship of the obtained sequences to modern cattle breeds from Africa, we constructed a phylogenetic tree using programs of the PHYLIP package and MEGA7 software (Fig 10). To prevent misinterpretation of the data due to differences in the sequence lengths, all sequences were trimmed to 273 bp. Both ancient sequences—ZS39 and DS3 were closely aligned to Menofi and Domiaty cattle breeds forming a monophyletic group, although obtained results need to be carefully interpreted. Bootstrap analysis returned low bootstrap number probably due to shortness of sequences and relatively low number of nucleotide substitutions. Genetic distance calculations are shown in Fig 11.

## Discussion

On the basis of the results it is possible to discuss two main economic aspects of life of El-Zuma/El-Detti people: agricultural nature of animal economy, trade directions and cultural contacts of the people buried in the tumuli fields at El-Zuma and El-Detti.

### Husbandry

Animal remains excavated from tumuli at El-Zuma and El-Detti and other archaeological sites in the vicinity, such as Kassinger Bahri [40], El-Sadda 1 [6, 14] and Tanqasi [41] consisted mostly of cattle and ovicaprine bones. It is interesting that the tumuli located on the left bank of the Nile (El-Sadda, Tanqasi) contained almost exclusively goat and/or cattle remains while animal material from the tumuli located on the right bank of the Nile (Kassinger Bahri, El-Zuma and El-Detti) consisted of sheep and/or cattle bones. Such zoological distribution might indicate agricultural nature of settlement activity in the area situated to the north of the Nile and its pastoral or semi-pastoral character to the south of the Nile. The goat can survive even very harsh environmental conditions, that is why some authors connect goats with pastoralism. The sheep is usually considered a farm animal, as a grazer it needs a better quality forage than goats [42]. This observation is significant in the light of the state of knowledge concerning the end of the Meroitic state in the 3rd or 4th century AD. There are two theories which attempt to explain what might have happened. The first thesis, which originated in the mid-20th century, is based on written sources [43, 44]. According to this theory, the end of the Meroitic state was caused by Noba invaders (nomads) from the west or south-west and the Aksumite Kingdom from the south-east, which established new small and insignificant kingdoms in the occupied territories. In other words, it assumes the sudden influx of people with different cultural patterns and a different lifestyle. The other hypothesis is based on archaeological findings and it is much more cautious as to the role of the Noba tribes in the fall of the state. Some researchers are inclined to accept that the long-term infiltration of the barbarian tribes changed the economic and political situation in the region and, in consequence, resulted in a gradual decline of the Meroitic state [13, 45]. This theory does not assume an exchange of the population, however. Archaeozoological materials from El-Zuma and El-Detti seem to indicate the continuity of rural settlement activity to the north of the Nile. The confirmation of this hypothesis by archaeozoological research is essential for further consideration of the economic and political situation in the region in the Early Makurian period. On the other hand, some archaeological sources indicate that the region to the south of the Nile was inhabited by semi-nomads. It is significant that P. Wolf and U. Nowotnick linked their discoveries at the Bayuda desert, located *c*. 2 km from the Nile Valley, with semi-pastoral lifestyle. The remains of the huts they excavated at several Post-Meroitic sites were probably used as seasonal camps [46].

Archaeozoological research shows the importance of the role of sheep among the people from the El-Zuma/El-Detti region. Sheep was commonly kept, as its bones were found in all the tumuli with animal offerings, also in the most poorly equipped ones. Its popularity as a food offering also indicates its lower value in comparison with cattle and transportation animals, such as camel, horse or donkey, which will be discussed further. Standard archaeozoological methods fail in most cases if we try to answer the question about the origin of the sheep herds. Due to the fact that juvenile sheep constituted the majority of such offerings it was possible to establish the height at the withers of only two individuals (one from El-Zuma and one from El-Detti). The animal from tumulus 14 at El-Zuma was 61.4 cm at the withers, the sheep from tumulus 4 at El-Detti– 67 cm. It seems that they were similar to the Dongola type, a woolly sheep described by H. Epstein [47], bred currently in the riverine area. According to this author, desert type individuals are bigger (70–90 cm). These results might suggest therefore that animals raised in the El-Zuma/El-Detti region in the Early Makurian period were of local, riverine breed, which should be confirmed with more extensive data. Unfortunately, there are no known studies concerning sheep from the that time in this region. It is, however, significant that the talus (GLl-29 mm) of sheep from the Neolithic site in El-Sadda 28 measured by M. Osypińska [48] was of a very similar size to the sheep taluses from El-Zuma (GLl-29.6–29.8 mm). They were certainly of different type from the sheep raised further north in Kerma a few thousand years earlier [49]. The average height at the withers of these sheep was 80 cm and they were described by the researchers as a hairy thin-tailed and long-legged primitive breed [49]. It is also interesting that the tall stature of the Kerman sheep was due to ‘particular development of the radius and the metapodials’ [49] while at El-Detti the withers height of an individual calculated from 3 different bones including the humerus (WH-63 cm), radius (WH-70.8 cm) and metacarpal (WH-66.6) differed only in a minor degree (especially the difference between the withers height calculated on the basis of the humerus and metacarpal was insignificant), which might suggest that the morphotype of El-Zuma/El-Detti sheep differed from this described for the Kerman sites.

Cattle morphotype might be described on the basis of macroscopic examination of skeletal remains on one condition: the sex of the individual must be known, because of sex dimorphism, which is reflected, among others, in the height at the withers. Size proportions of some bones, such as metacarpals and metatarsals or the shape of horn-cores are also helpful in establishing the sex. Unfortunately, these elements are practically absent in the material from El-Zuma and El-Detti. Another problem is a very high share of juvenile cattle remains in the material from both sites, which makes measurements useless as bones of young animals still grow. All cattle remains discovered at El-Detti were in a bad state of preservation and it was impossible to take the length measurements. Therefore, only comparison of results of aDNA analysis could provide information about cattle kept in this region. Only in one case of a female from tumulus 4 at El-Zuma it was possible to establish the height at the withers. The result between 120.4 cm and 124.7 cm indicates a medium-sized individual. Additionally, no thoracic vertebrae with bifid dorsal spines were found at the sites, which might suggest that the cattle kept in the El-Zuma/El-Detti region was of taurine type.

Indigenous population of modern African cattle includes three groups: taurine, indicine, and hybrid breeds (together more than 150 different breeds) [50]. This diversity is an effect of a few thousand years history of hybridization [50, 51, 52]. Geographic distributions of genetic markers made for modern African cattle breeds helped to compare the animals that were raised in the El-Zuma/El-Detti microregion with cattle breeds from other African regions and construct a phylogenetic tree. It is interesting that sequences obtained from both analyzed sites were closely aligned to Menofi and Domiaty cattle breeds. Menofi (also called Baladi) and Domiaty (Damietta) are two humpless Egyptian cattle breeds from the Nile Delta and of the Near East origin [33, 53]. Surprisingly, ancient samples were not so closely related to other African cattle breeds, thus El-Detti and El-Zuma animals might have been of Egyptian origin and they did not hybridize with African breeds. On the other hand, we should bear in mind that zebu (*Bos indicus*) introduction into this part of the African continent might have been connected to the Arabic trade activity which took place about the 7th century AD [51, 52]. The absence of indicine component at El-Zuma and El-Detti might be therefore an important marker for confirmation of this theory. Nevertheless, the results need to be interpreted with caution due to the shortness of sequences and low yield of DNA amplification, which reflects its poor quality. More detailed analysis, involving Next Generation Sequencing methods would be necessary to confirm and expand the knowledge regarding the origin of cattle from El-Zuma and El-Detti archaeological sites.

### Trade and cultural contact directions

The results delivered by archaeological research supplemented with genetic data of cattle remains indicate possible directions of trade and interregional contact maintained by the people from the El-Zuma/El-Detti microregion in the Early Makurian period. On the basis of the pottery found in the graves at El-Zuma it can be concluded that such interregional contacts took place [2, 54]. The main direction of these contacts was the north. Import of pottery from the Lower Nubia and Egypt is testified in the richer tumuli of El-Zuma as well as at other sites of the Early Makurian provenience, such as Hammur Abbassiya or Kassinger-Bahri [13]. It seems that not only trade exchange was an important objective of these contacts. There are a lot of indications that might also suggest deriving cultural patterns from the North, among others—a specific kind of animal offerings discovered in the tunnels of tumuli 1 and 4 at El-Zuma where the whole animals were probably placed in the graves in the course of an unknown funerary ritual. The burials excavated at Balana and Qustul could be analogies to such rituals [55], with a slightly earlier chronology than El-Zuma site (the so called X-group). The tumuli at Balana and Qustul were usually bigger, with more complex underground structures and probably also better equipped, but the pattern seems to be the same: cattle, sheep, horse, donkey and camel, complete or almost complete skeletons found in these graves were certainly not the remains of food offerings—there were no post-consumption marks on the bones and the bones themselves were in most cases articulated. There is no certainty whether the animal carcasses from El-Zuma were deposited intact in the graves—excavated bones were broken into pieces and mixed with earth and other finds, but the presence of head elements and extremities suggests that it might be the case. Only very few bones of sheep and cattle from these graves bore post-consumption marks, such as cutting and filleting marks, which implies that two different rituals took place during the funeral: one connected with food offerings for the dead, discovered at Balana and Qustul, as well as at El-Zuma and El-Detti, and the other reflected in the whole animal carcasses located on ramps in the case of Balana and Qustul and in the tunnels in the case of El-Zuma. These are not the only analogies to Balana and Qustul. There are others, for instance metal jewellery or game pawns made of ivory found in tumulus 7 at El-Zuma. The only known analogy to these latter finds in this part of Africa is a set of game pawns discovered at Qustul ([55] plate 87).

The results of aDNA analysis of cattle remains from El-Zuma and El-Detti also directs us to the North. It is, however, impossible to establish whether the animals from these Early Makurian sites belonged to the herds kept in the region for some time, perhaps even centuries, or were brought there as objects of trade in the lifetime of the people who were buried in the graves at El-Zuma and El-Detti.

The presence of transportation animals in the tombs was probably connected with transit trade routes, perhaps controlled by the local rulers [13]. Profits from trade might have paved the way to their wealth.

## Conclusions

The number of the graves (only 29 in El-Zuma tumuli field and 44 in El-Detti tumuli field) cannot reflect the number of people who lived in the region between mid-5th and mid-6th century AD, therefore we should expect that both tumuli fields contained only the graves of well-to-do people in general. It is also remarkable that further social stratification of the people buried in the tumuli at El-Zuma and El-Detti is visible in the size and type of structure of the features as well as in the equipment of the graves, including animal offerings. It seems that only people of higher position could afford to keep cattle which might be confirmed, among others, by almost complete lack of such remains in poorer equipped tumuli of type 3. It is interesting that mostly young animals were chosen for the ritual purposes. Veal is a very highly valued type of meat not only because of its quality, taste and amount but also because slaughtering a calf deprived its owners of some secondary products such as traction or milk in the future. Therefore, the rule that veal/beef offerings were commonly placed in tumuli of type 1 and 2 and absent or almost absent from tumuli of type 3 seems to confirm the thesis, which indicates that only the elite was buried in the tumuli of type 1 and 2 at El-Zuma. On the other hand, the scarcity of tombs of type 2 and almost complete absence of tumuli of type 1 at El-Detti and other tumuli fields in the region [13] seems to demonstrate the special position of the people who lived in the vicinity of El-Zuma cemetery. The only two excavated tumuli of type 1 outside El-Zuma site were located at Hammur Abbassiya [56]. It is also significant that bones of transportation animals (camel, horse, donkey) were found only in the richest graves at El-Zuma, such remains were absent entirely at the much poorer cemetery at El-Detti.

The equipment of the tumuli at El-Zuma indicates the existence of an agricultural society that mostly kept sheep herds, ruled by wealthy cattle herd and sheep keepers, who might have also been merchants or those who profited from the trade directly or indirectly. The elite could spend the time hunting, which is testified by gazelle remains in tumuli 1 and 4 and also indirectly by finds such as archer’s looses and arrows. These wealthy people could also play board games in their leisure time, which is confirmed by the finds of game pawns in tumulus 7. This social stratification is not that evident on the basis of the material from the El-Detti tumuli field, which might indicate that the true power and wealth were elsewhere.

## Supporting information

S1 Table List of samples. (XLSX)
